# Timing of complementary feeding and associations with maternal and infant characteristics: A Norwegian cross-sectional study

**DOI:** 10.1371/journal.pone.0199455

**Published:** 2018-06-27

**Authors:** Christine Helle, Elisabet R. Hillesund, Nina C. Øverby

**Affiliations:** Department of Public Health, Sport and Nutrition, Faculty of Health and Sport Sciences, University of Agder, Kristiansand, Norway; Indiana University Bloomington, UNITED STATES

## Abstract

Norwegian Health authorities recommend solid food to be introduced between child age 4–6 months, depending on both the mother´s and infant’s needs. The aim of this paper is to describe timing of complementary feeding in a current sample of Norwegian mother/infant-dyads and explore potential associations between timing of introduction to solid foods and a wide range of maternal and infant characteristics known from previous literature to influence early feeding interactions. The paper is based on data from the Norwegian randomized controlled trial *Early Food for Future Health*. In 2016, a total of 715 mothers completed a web-based questionnaire at child age 5.5 months. We found that 5% of the infants were introduced to solid food before 4 months of age, while 14% were not introduced to solid food at 5.5 months of age. Introduction of solid food before 4 months of age was associated with the infant not being exclusive breastfed the first month, receiving only formula milk at 3 months, the mother being younger, not married/cohabitant, smoking, less educated and having more economic difficulties. Not being introduced to solid food at 5.5 months was associated with the infant being a girl, being exclusive breastfed the first month, receiving only breastmilk at 3 months, the mother being older, married and having 3 or more children. This study shows that there are still clear socioeconomic differences regarding timing of complementary feeding in Norway. Infants of younger, less educated and smoking mothers are at higher risk of not being fed in compliance with the official infant feeding recommendations. Our findings emphasize the importance of targeting socioeconomically disadvantaged mothers for support on healthy feeding practices focusing on the infant`s needs to prevent early onset of social inequalities in health.

## Introduction

Introducing the first solid food constitutes an important milestone in the infant's development. The World Health Organization (WHO) recommends exclusive breastfeeding during the first six months of life, followed by a gradual introduction of food in parallel with continued breastfeeding [1, 2]. These recommendations apply to all countries and populations, regardless of economic status or developmental level [3]. The European Society for Pediatric Gastroenterology, Hepatology and Nutrition (ESPGHAN) Committee on Nutrition supports the recommendations of exclusive breastfeeding for the first six months [4], but adds that complementary feeding should not be introduced before 17 weeks (4 months) and not later than 26 weeks (6 months) [5].

In most European countries solid food is introduced before child age 6 months [6]. The latest Norwegian national cross-sectional study on infant nutrition from 2013 [7] showed that 7% of the infants were introduced to solid food before 4 months of age, while only 21% of the infants were introduced to their first solid food at six months of age. Since 2016 the Norwegian Health authorities recommend that infants should be exclusively breastfed for the first 4 to 6 months depending on the mother´s and infant’s needs, followed by a gradual introduction of food in parallel with continued breastfeeding. At child age six months, introduction of solid food is recommended independent of breastfeeding status. Infants who have been exclusively breastfed will now need supply of iron-rich foods to ensure adequate growth and development [8].

Valid reasons for introducing solid food from 4 months of age may be that the infant shows insufficient weight gain or growth, seems hungry after frequent breast-/formula meals or that the child shows clear interest in other foods [8]. These reasons are linked both to infant characteristics like age and weight, but also to the mothers’ ability to interpret infant feeding cues like hunger, satiety and interest in food. Early feeding decisions have a bidirectional relationship [9], as infant characteristics may influence maternal responses and infants may respond differently to parental behaviors. In addition, maternal characteristics may influence her perception of infant feeding cues. Previous research has shown higher levels of maternal responsiveness to child fullness cues among mothers with lower BMI [10]. Altogether, this highlights the complex and dyadic nature of early feeding interactions.

There is a well-established association between milk feeding mode (breast milk, formula milk or mixed feeding) and the timing of introduction to complementary food [11–13]. In Denmark, mothers who were partially or not breastfeeding at child age 2 and 4 months, introduced solid food earlier than mothers who fully breastfed their infants [14].

A Cochrane review from 2016 found no evidence to disagree with the current international recommendation that healthy infants should be exclusively breastfeed for the first six months [15]. However, mothers provide various health-related reasons for introducing solid food earlier. In a qualitative exploration of factors influencing first-time Australian mothers`introduction of complementary food, the mothers`main beliefs were that introducing food would assist the infants weight gain, improve sleeping patterns and increase enjoyment at mealtimes [16]. A qualitative systematic review including papers from the US, Europe and Australia, found that mothers used food to influence infant growth, contentment and sleep [17]. A study from the UK found that solid food often was introduced to settle behavior and encourage sleep [18]. In another study, mothers of infants with perceived difficult temperament reported less awareness of infant hunger cues, more use of food to calm, and higher concern about over- and underweight [19]. Concerns about growth and development may apply especially to premature born children, and previous research has shown that preterm infants are significantly more likely to be early introduced to solid food [20].

Maternal demographic factors such as age, parity, education and socioeconomic status as well as behavioral factors like smoking, are known to influence early feeding decisions [20–25]. In Norway the number of smokers is decreasing, while there is an increasing number of young men and women using snus (powdered tobacco). In 2013, 23% of young women (16–24 years) used snus daily or occasionally [26]. Maternal mental and physical health characteristics may also influence feeding style and practices. Maternal symptoms of anxiety and depression have been associated with a lower extent of breast feeding [27], increased use of control and pressure to eat [27–30], and a less wholesome and a more unhealthy diet [31]. The association between maternal anxiety or depression and early introduction of solid food is more unclear. While maternal mental health symptomatology has been associated with early introduction to complementary food in some studies [32, 33], another study found that this relationship was not significant after adjusting for potential confounders [34]. A high maternal BMI has in previous studies been negatively associated with initiation and duration of breastfeeding [35, 36], and a higher pre-pregnancy BMI has been negatively correlated with the timing of solid food introduction [9]. However, a Danish study found no association between higher maternal pre-pregnancy BMI and earlier introduction to solid food if the infant was fully breastfed past five weeks [37]. They concluded that further research was needed to clarify the association between breastfeeding, maternal BMI and timing of solid food introduction.

The aim of this paper is to describe the timing of introduction to complementary food in a current Norwegian population of mother-infant dyads. We explore possible associations between timing of introduction to solid food and a broad range of maternal and infant characteristics known from previous literature to be associated with early feeding interactions. The paper is based on data from the Norwegian study *Early Food for Future Health*, a randomized controlled trial in which an e-health intervention aiming to foster protective parental feeding behavior and promote early healthy food habits in the weaning period has been developed and will be evaluated [38]. The study sample consists of 715 Norwegian mother-infant dyads, all infants born in 2015 and 2016.

## Methods

### Study design and participants

In springtime 2016, parents from all over Norway were eligible to participate in the study *Early Food for Future Health* if they had a 3–5 months old infant born after gestational week 38, were literate in Norwegian and were responsible for providing food to their infant. The parents were recruited by posting an information site and a short video on social media (Facebook). In addition, an email with link to the study’s homepage was sent to all the Norwegian municipality`s child health centers to inform the public health nurses about the study. Parents consented to participate on the study`s homepage [39].

**Fig 1:**
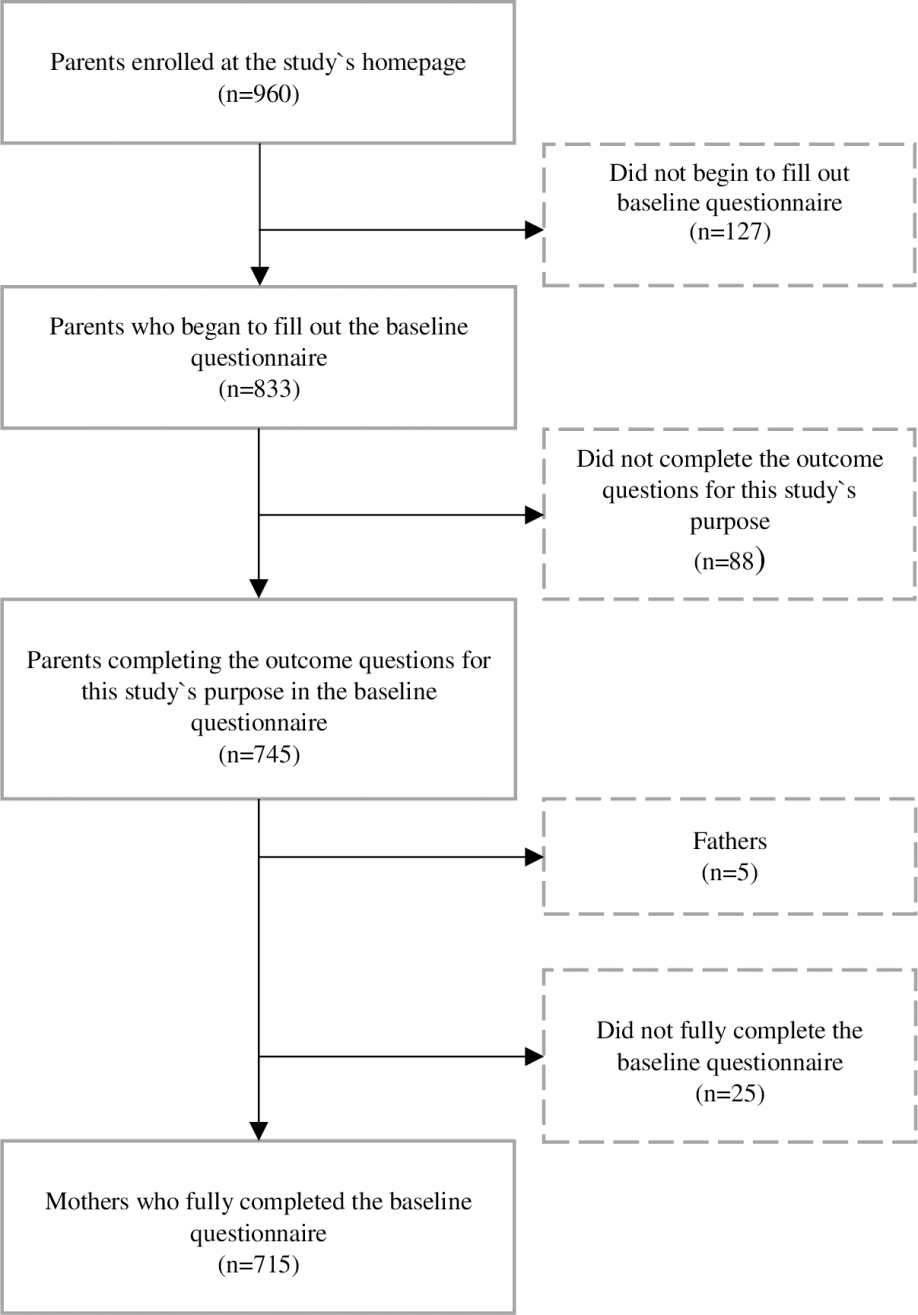
Flowchart of participants.

A total of 960 parent-infant dyads registered for participation. The flowchart of participants is described in Fig 1. The self-administered, web-based questionnaire was distributed to the parents at child age 5 months and had to be filled in and submitted before child age 6 months. A total of 833 parents started filling in the questionnaire. Of these, 745 parents (740 mothers and five fathers) responded to the outcome questions for this paper (timing of solid food introduction). Because the large majority of respondents were mothers, the fathers were excluded from the present study. In total 715 mothers completed the full questionnaire. We chose to include all mothers/infants regardless of gestational age, as the intervention was designed to be used also by this grouping.

We included all available responses for each of the outcome variables in this study, making the total number of participants in the analyses vary between 715 and 740. All data were collected between March and August 2016. The Norwegian Centre for Research Data evaluated and approved the study. Informed consent from all participating parents was obtained upon registration. Details regarding recruitment and study design have previously been published [38].

### Variables and measures

#### Outcome measures; timing of introduction to complementary food

We use the terms “complementary food” or “solid food” to describe any firm, soft or liquid food or drink other than milk and water [11]. A Food Frequency Questionnaire (FFQ) was developed for the Early Food for Future Health study to assess daily food consumption, breastfeeding duration and age at introduction of solid food. The questionnaire was based on questionnaires from a Norwegian national dietary survey in 6 months old children [40] and the large population based Norwegian Mother and Child Cohort Study (MoBa) from the Norwegian Institute of Public Health [41], and was not validated.

Parents responded to questions concerning time of introduction to homemade and industrial made complementary food as “How old was your infant when he/she first was introduced to these foods?” *(porridge—industrial and homemade*, *mashed vegetables—industrial and homemade*, *mashed fruits—industrial and homemade*, *meals with red/white meat or fish—industrial and homemade*, *bread)* Response alternatives were given in months.

Questions about food, breastmilk and other liquids the first six months were asked as follows: “What kind of food types and drinks have the infant received the first 6 months of life? Tick the box for each month (0, 1, 2, 3, 4, 5, 6) the infant has received the listed food types and drinks” *(Breastmilk*, *formula*, *water*, *juice*, *baby porridge*, *dinner*, *fruits/berries)*.

We constructed the outcome variable “Timing of solid food introduction” by summing whether the child had been introduced to either *juice*, *baby porridge*, *dinner* or *fruits/berries* during a selected period of time. Milk (breastmilk/formula) and water were excluded from the calculation of introduction-time of solid food. The infants were divided into four groups depending on first-time introduction of complementary food: Infants introduced before 4 months of age, infants introduced between 4 and 5 months of age, infants introduced between 5 months and 5.5 months of age, and infants who had *not* been introduced to solid food at 5.5 months of age. We only had data up to 5.5 months of age because of the deadline for submission of the questionnaire. The deadline was set in advance due to the initiation of the study’s intervention at child age 6 months. An infant would be classified as “introduced to solid food between 4 and 5 months of age” if s/he had been introduced to either *juice*, *baby porridge*, *dinner* or *fruits/berries* for the first time in month 4.

#### Infant characteristics

Infant gender, gestational age in weeks *(<38*, *≥38)*, birth weight (g) and length (cm) were reported by the mothers. The mothers also reported date of visit and infant weight and length as measured at the child health center at 3 and 5 months of age. Mothers had the opportunity to omit responding if the child had not visited the health center at these timepoints. Z-scores for BMI-for-age at birth, three and five months were calculated using the software program WHO Anthro version 3.2.2. (Department of Nutrition, World Health Organization, Geneva, Switzerland) and macros [42]. We calculated the differences in BMI z-scores *(zBMI-change)* from birth to three months of age and from three to five months of age as a measure of the infants`weight gain.

Infant temperament was assessed using a short version of the fussy/difficult subscale from *The Infant Characteristics Questionnaire—6 Months Form* (ICQ-6) [43]. Mothers were asked to report the extent to which they agree in statements concerning their infant’s temperament, e.g. “The child is easily upset”. Parents ranked seven items on a 7-point scale *(completely disagree*, *disagree*, *disagree somewhat*, *indifferent*, *agree somewhat*, *agree*, *agree completely)*, indicating the level of perceived difficulties in dealing with the described behavior. An average score ranging from 1 to 7 was calculated, with higher scores reflecting greater infant difficulties. Cronbach`s α for this scale in our sample was 0.76. The short form of this questionnaire has previously been used in the population-based Norwegian MoBa-study [44].

Sleeping problems were measured with two different questions: 1) “Currently how often does your child usually wake up during the night?” with four response alternatives *(3 or more times every night*, *once or twice every night*, *a few times a week*, *seldom or never)*. These were dichotomized into *3 or more times every night* and *less than 3 times every night*. 2) “How many hours in total does your child sleep per 24 hours?” with five response alternatives *(less than 8 hours*, *8–10 hours*, *11–13 hours*, *13–14 hours and more than 14 hours)*. In the current sample, more than 80% of the infants slept 13 hours or more per 24 hours. The response categories were dichotomized to *less than 13 hours* and *13 hours or more*.

#### Milk-feeding mode

Milk-feeding mode at the following two time-points was included: 1) Exclusively breastfed first month (month 0), *yes* or *no*. Being exclusively breastfed is defined as receiving only breastmilk and not being introduced to any other liquids or food except vitamin supplements [45]. 2) At infant age three months, milk-feeding mode were categorized into *only breastfed*, *breastfed and formula-fed* and *only formula-fed* regardless of whether solid food was introduced or not.

#### Maternal characteristics

Maternal sociodemographic variables included age *(years)* computed from self-reported birth-date, marital status (categorized into *married*, *cohabitant* and *not married/cohabitant*), number of children (*one child*, *two children*, *three or more children*) and native language (dichotomised into *Norwegian* vs. *not Norwegian*).

Socioeconomic status was measured with the following questions: 1) “What is your highest completed education?” Education level was recorded on a 7-category scale and subsequently recoded into high vs. low education level *(college/ university education* vs. *no college/university education)*. 2) “What is (was) your main activity (before maternity leave)?” The answer had 10 response categories, these were recoded into *working full-time*, *working part-time*, *student*, *not working*. 3) Degree of urbanization were reported in four categories: ≤ 4999, 500–1499, 15000–49999, ≥ 50000 inhabitants. 4) Economy was assessed by two questions: “Are you able to pay an unforeseen expense of 3000 NOK?”, with the response options *yes*, *no* and *maybe*. These were recoded into *yes* and *no/maybe*. The last question was if they had experienced difficulties paying their rent, food or transportation during the last six months. Possible responses were *no-never*, *yes-seldom*, *yes-sometimes* and *yes-often*. These were subsequently recoded into *no difficulties/difficulties*.

Maternal BMI (kg/m2) was calculated using self-reported height and weight at the time of completion of questionnaire. WHO-guidelines were used to classify participants as underweight (BMI < 18.5 kg/m^2^), normal weight (BMI 18.5–24.9 kg/m^2^), overweight (BMI 25.0–29.9 kg/m^2^), and obesity (BMI ≥ 30 kg/m^2^ [46]. Only a small percentage (1.5%) of the mothers were categorized as underweight, the BMI-categories were therefore classified into *underweight/normal weight* (BMI < 25), *overweight* (BMI 25.0–29.9) and *obesity* (BMI ≥ 30).

Self-perceived physical health was asked as follows: “All in all; How will you characterize your physical health?”, with four response categories *(very good*, *good*, *bad*, *very bad)* dichotomised into *good* and *bad* health. Use of self-reported health in Norwegian surveys has shown good credibility in reporting differences in health [47].

Maternal symptoms of anxiety and depression were assessed using a short version of the The Hopkins Symptoms Checklist (SCL-90) [48]. This is is a well-established psychometric instrument, and the short version used in this study (SCL-8) has previously been used in the Norwegian MoBa-study [49]. Symptoms are mapped as “Have you been bothered by any of the following during the last two weeks?” Four items are capturing depressive symptoms (e.g. *“feeling hopeless about the future”*), and four questions are tapping anxiety symptoms (e.g. “*feeling fearful”*). Each question includes four categories of response *(not bothered*, *a little bothered*, *quite bothered*, *very bothered*) rated 1 to 4, with higher scores reflecting more severe symptoms. The average item score is computed by adding the item scores and dividing the sum score on the number of items. Cronbach`s α for this scale in our sample was 0.83. The SCL-8 total score was dichotomized following the recommended cut-off at 2.00 [50, 51] into a *high* and a *low* score.

We included smoking and use of snus as lifestyle variables. Response categories for “Do you smoke?” and “Do you use snus?” were *no—never*, *no—quit*, *yes—not daily*, and *yes—daily*. These were recoded into *yes* and *no*.

### Statistical analysis

Associations between timing of introduction of complementary food and each of the potential infant- and mother predictor variables were assessed separately using chi-square tests for categorical variables. The Fisher exact test were used for the variables *marital status*, *able to pay unforeseen expense*, *SCL-8 score high/low* and *smoking* because of small sample sizes. One-way analysis of variance (ANOVA) was used to compare means for continuous variables.

Binary logistic regression analyses were performed to assess the relationship between the individual infant- and mother characteristics and the dependent variables *early* (before four months of age) and *later* (after 5.5 months of age) introduction to solid food. Separate models were used to examine infant and maternal factors associated with early/late introduction, respectively. Potential violations of multicollinearity assumptions were tested by treating the categorical values as continuous in a linear regression, and collinearity diagnostics were performed.

All the infant and maternal predictor variables were included for initial exploration in univariate tests. We used zBMI-change 0–3 months for both outcomes (early- and later introduction), and zBMI-change 3–5 months only for the outcome later-introduction. Univariate tests with p-values less than 0.2 were selected for use in the logistic regression models. When adjusting the models, we excluded successively the variable with the highest p-values until all remaining variables in the final models were significant. There are different variables included in the different final models, as only the variables with p-value less than 0.05 were selected for each final model. The infant`s sex and the maternal age were included in the models regardless of their individual statistical significance because of their explorative value.

The outcome variables (*early* and *later* introduction to solid food) are categorical. The predictor variables are either dichotomous, categorical or continuous. A two-tailed 5% level of significance was used, and results are presented as adjusted odds ratios (OR) with 95% confidence intervals. All analyses were performed with the statistical software package IBM SPSS Statistics version 24.0 (IBM Corp., Somers; NY, USA).

## Results

### Characteristics of study sample

Mother and infant characteristics are shown in Table 1. The infants`mean age at completion of the questionnaire was 5.5 months (22.3 weeks ± 1.5 week). Maternal age ranged from 18 to 44 years (mean 30.4) The mothers who reported not having Norwegian as their mother tongue (7.3%) constituted a heterogeneous group, with the majority speaking another Scandinavian or European language. Most of the participating mothers had higher education at university/college-level (80.7%), had been working full time before pregnancy (80.6%) and were married or cohabitant (97.9%). For just over half of the mothers (56.8%), this was their first child. The maternal mean BMI was 24.9. The majority of mothers (62.4%) lived in urban areas with more than 15 000 inhabitants.

**Table 1:** Characteristics of the participating mother-infant dyads (n = 740^1^).

Characteristics		Values	Mean (SD) or %*
**Infants**			
	Sex	Boy	50.9
		Girl	49.1
	Gestational age (weeks)	> = 38	90.3
		<38	9.7
	Birth weight (g)		3578 (496)
		>3500	55.3
		2500–3500	43.1
		<2500	1.6
	Birth length (cm)		50.3 (2.2)
	Weight at 3 months of age (13,0 (0,8) weeks)		6307 (778)
	BMI z-score at 3 months of age		-0.17 (1.01)
	Weight at 5 months of age (21,7 (0,93) weeks)		7589 (920)
	BMI z-score at 5 months of age		0.05 (0.99)
**Mothers**			
	Age (years)		30.4 (4.4)
	Norwegian as native language (self-reported)		92.7
	Parity	Primipara	56.8
		Multipara	43.2
	Marital status	Married	39.6
		Cohabitant	58.3
		Not married/cohabitant	2.1
	Education	Lower secondary school or less	1.8
		Upper secondary school	16.6
		College/university (< = 4 years)	36.8
		College/university (>4 years)	43.9
		Other	0.8
	Main activity (before pregnancy)	Working fulltime	80.6
		Working part-time	6.7
		Student	7.3
		Not working	5.3
	Degree of urbanization	+50 000	41.8
		15 000–49 999	20.6
		5000–14 999	25.4
		- = 4999	12.1
	Maternal BMI (kg/m^2^) (after pregnancy)		24.9 (4.4)

^**1**^ The total number of participants in the analyses vary between 715 and 740 depending on the outcome

* Valid percentages for categorical variables, and means with SD for continuous variables

Of the participating infants, 90.2% were born in or after gestational week 38, their birth weight ranged from 1920 g to 5270 g (mean 3578 g). The mean birth weight in our sample was slightly above the national average (3481 g) [52], and the proportion of boys/girls close to the national average.

### Timing of different food types

**Fig 2:**
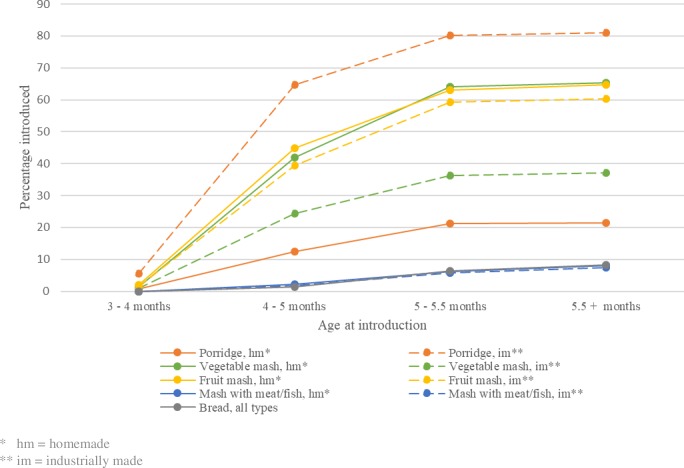
Cumulative proportion of infants introduced to different food types.

The cumulative proportion of infants introduced to the various food types is shown in Fig 2. None of the mothers reported introduction of solid food before child age three months. The most frequent food to be first introduced across all age groups was industrially made porridge. At child age 5.5 months, 81% of the infants had been introduced to industrially made porridge while 21.4% were introduced to homemade porridge. On the other hand, infants were more often introduced to homemade than industrially made vegetable mash (65.4% vs. 37.2%) and fruit mash (64.8% vs. 60.4%). The least frequent food types to be introduced were mash with meat/fish (both homemade and industrially made) and bread. Less than 10% of the infants were introduced to these food types at age 5.5 months.

### Timing of complementary feeding according to infant and maternal characteristics

Table 2 shows the characteristics of infants and mothers stratified into four groups according to timing of introduction to complementary food. Regarding infant characteristics, a significant difference was found between the groups with respect to the gender of the child (p = 0.04), with more girls than boys *not* being introduced to solid food at 5.5 months of age. There was no significant difference between being born before/after gestational week 38 and timing-of-introduction.

**Table 2:** Infant and maternal characteristics according to timing of introduction to solid food (N tot = 740)^1^.

			Introduction to solid food								
			Before 4 months		Between 4 and 5 months		Between 5 and 5.5 months		Not introduced to solid food at child age 5.5 months		
			N = 37 (5.0%)		N = 430 (58.1%)		N = 168 (22.7%)		N = 105 (14.2%)		
Variable		Values	N	Mean (SD)* or % ^P^	N	Mean (SD)* or % ^P^	N	Mean (SD)* or % ^P^	N	Mean (SD)* or % ^P^	P value ^§^
**Infant factors**											
	Sex	Girl	14	37.8	200	46.5	86	51.2	63	**60.0**	**0.040**
		Boy	23	62.2	230	53.5	82	48.8	42	**40.0**	
	Gestational age in weeks	≥38	34	91.9	386	89.8	156	92.9	92	87.6	0.502
		<38	3	8.1	44	10.2	12	7.1	13	12.4	
	Birthweight (g)		37	3611 (534)	430	3551 (489)	168	3640 (504)	105	3580 (493)	0.251
	zBMI-change from birth to 3 months		33	-0.58 (1.46)	390	-0.71 (1.15)	157	-0.62 (1.11)	94	-0.49 (1.15)	0.42
	zBMI-change from 3 to 5 months		25	0.38 (0.56)	277	**0.26** (0.61)	120	**0.07** (0.57)	62	0.18 (0.75)	**0.021**
	Sleeping difficulties	Wakes 3 or more times a night	10	27.0	141	32.8	59	35.1	41	39.0	0.500
		Sleeps less than 13 hours per day/night	6	16.2	85	19.8	37	22.0	16	15.2	0.537
	Temperament	Fuzzy/difficult temperament subscale score	35	2.42 (0.92)	419	2.50 (0.83)	164	2.44 (0.91)	101	2.43 (0.82)	0.780
	Feeding factors										
	Exclusive breastfed first month	No	27	**73.0**	144	33.5	42	**25.0**	28	26.7	**<0.001**
	Breastfed or formula-fed at age 3 months	Only breastfed	12	**37.5**	278	**66.0**	144	**87.3**	91	**91.9**	**<0.001**
		Breastfed and formula-fed	5	15.6	96	**22.8**	14	**9.1**	8	**8.1**	
		Only formula-fed	15	**46.9**	47	11.2	6	**3.6**	0	**0.0**	
**Maternal Factors**											
	Age in years		34	**28.8** (5.3)	393	30.3 (4.2)	155	30.6 (4.4)	89	**31.4** (4.3)	**0.018**
	Number of children	1	22	64.7	259	**62.0**	83	50.9	42	**41.6**	**<0.001**
		2	8	23.5	118	28.2	58	35.6	28	27.7	
		≥3	4	11.8	41	**9.8**	22	13.5	31	**30.7**	
	Marital status	Married	5	**14.7**	167	39.9	57	35.0	55	**54.5**	*<***0.001** <0.001**
		Cohabitant	26	**76.5**	244	58.2	104	63.8	44	**43.6**	
		Not married/cohabitant	3	**8.8**	8	1.9	2	1.2	2	2.0	
	Sociodemographic variables										
	Education	Low	12	**35.3**	77	18.5	24	14.7	19	19.4	**0.047**
	Economy										
	Unforeseen expense	Not/maybe able to pay	4	**11.8**	21	5.0	2	**1.2**	4	4.0	**0.035** 0.024**
	Running expenses	Problems	8	23.5	62	14.9	20	12.3	20	19.8	0.208
	Maternal health factors										
	Maternal BMI-categories	Under-/ Normal weight	17	50.0	252	60.3	109	66.9	57	56.4	0,521
		Overweight	11	32.4	110	26.3	35	21.5	30	29.7	
		Obese	6	17.6	56	13.4	19	11.7	14	13.9	
	Physical health (self-reported)	Poor health	4	11.8	44	10.6	17	10.4	10	9.9	0.992
	Mental health SCL8, score	High	1	2.9	23	5.5	7	4.3	3	3.0	0.689 0.775**
	Life-style factors										
	Smoking	Yes	5	**14.7**	14	3.3	7	4.3	1	1.0	**0.003** 0.012**
	Use of snus	Yes	3	8.8	29	6.9	7	4.3	1	**1.0**	0.083 0.049**

^**1**^ The total number of participants in the analyses vary between 715 and 740 depending on the outcome

*Valid percentages for categorical variables, and means with SD for continuous variables

^**§**^ The groups that differ significantly are marked in bold

** Fisher`s Exact Test

^P^ Within group percentages

Dates for visits at the child health center with corresponding measured weight and length were available for 674 infants for 0 and 3 months of age and 484 infants for 3 and 5 months of age, respectively. We found no significant difference between the groups regarding zBMI-change 0–3 months. For zBMI-change 3–5 months, we found that infants in the two earliest timing-of introduction groups had the highest values, but the difference reached significance only for the group introduced to solid food between 4 and 5 months of age (p = 0.021). When comparing change in weight-for-age z-scores (WAZ) from 3–5 months between the groups (not shown), we found that the WAZ-score for the early introduction group was significantly higher (0.44 ± 0.55) compared to the score for the group introduced to solid food between 5–5.5 months (0.06 ± 0.39) and the score for the later introduction group (0.12 ± 0.55), p*<*0.001.

There were also significant differences between the groups depending on whether the infant was exclusively breastfed the first month of life (p<0.001) and on milk-feeding mode at three months of age (p<0.001). Infants who were introduced to solid food before four months of age, were less often exclusively breastfed the first month of life and were more often receiving only formula milk as milk type at three months of age. There were no significant differences for birth weight, gestational age, sleeping difficulties or maternal perceived infant temperament.

Regarding maternal characteristics, significant differences between the different timing-of-introduction groups were found for age (p = 0.018), parity (p = 0.001), marital status (p<0.001), education (p = 0.047), economic difficulties (p = 0.024), smoking (p = 0.012) and use of snus (p = 0.049). Mothers introducing solid food before four months of age, were characterized by more frequently being younger, not married/cohabitant, smoking, having lower educational level and more economic difficulties. There was also an observed trend with a higher proportion of overweight and obese mothers in this group, but the difference did not reach significance. The group of mothers who had *not* introduced their infants to solid food at child age 5.5 months, were characterized by more frequently being older, married, having more than one child and not using snus. There were no significant differences between the groups with respect to self-perceived physical and mental health.

Infant and maternal characteristics that independently predicted early or later introduction to solid food are presented in Table 3. Infants who were not exclusively breastfed during the first month of age and infants who were given only formula as type of milk at three months of age, had higher odds of being introduced to solid food before four months of age compared to infants who were exclusively breastfed first month of life and given only breastmilk at three months of age. Girls had higher odds of later introduction to solid food compared to boys. Infants who were given a combination of breastmilk and formula at three months of age had lower odds of later introduction to solid food compared to infants who were given breastmilk as milk type at three months of age. In the group of infants who were not introduced to solid food at 5.5 months, none were given only formula.

**Table 3:** Associations between timing of introduction to solid food (early and later) and infant- and maternal characteristics estimated by multivariate logistic regression.

		**Early introduction to solid food** ^**§**^ **N = 37**		**Later introduction to solid food** ^**¶**^ **N = 105**	
**Characteristics**		**OR**	**95% CI**	**OR**	**95% CI**
**Infant factors**					
Gender					
	Girl	1.00		1.00	
	Boy	1.91	0.88–4.15	0.62*	0.40–0.96
Feeding characteristics					
	Exclusively breastfed first month	1.00			
	Not exclusively breastfed first month	3.38**	1.46–7.65		
Milk-feeding mode at 3 months of age					
	Breastmilk	1.00		1.00	
	Breastmilk and formula	1.29	0.43–3.83	0.34**	0.16–0.72
	Formula	8.13***	3.39–19.53	0.00	0.00
^§^ Adjusted for infant gender, exclusive breastfeeding first month of age and milk-feeding mode at three months of age. ^¶^ Adjusted for infant gender and Milk-feeding mode at 3 months of age. Separate models are presented in this table with different covariates included, this explains the open spaces *p<0.05 **p <0.01 ***p<0.001					
		**Early introduction to solid food** ^**§**^ **N = 37**		**Later introduction to solid food** ^**¶**^ **N = 105**	
**Characteristics**		**OR**	**95% CI**	**OR**	**95% CI**
**Maternal factors**					
Age		0.93	0.85–1.01	1.02	0.96–1.08
Marital status					
	Married	1.00		1.00	
	Cohabitant	3.28*	1.23–8.72	0.50**	0.31–0.80
	Not married/cohabitant	7.83*	1.51–40.62	0.76	0.16–3.62
Number of children					
	1			1.00	
	2			1.28	0.74–2.23
	≥3			3.39***	1.83–6.27
Smoking status					
	Not smoking	1.00			
	Smoking	4.21*	1.40–12.72		

^§^ Adjusted for maternal age, marital status and smoking.

^¶^ Adjusted for maternal age, marital status and number of children.

Separate models are presented in this table with different covariates included, this explains the open spaces

*p<0.05

**p <0.01

***p<0.001

Mothers who were cohabitant or not married/cohabitant had higher odds of early introducing their children to solid food compared to mothers who were married, this also applied to mothers who smoked compared to non-smoking mothers. Mothers who were not married had lower odds of later introduction to solid food compared to married mothers, but this was only significant for the cohabitant group of mothers. Mothers with tree or more children had higher odds of later introduction to solid food compared to mothers with one child.

## Discussion

The majority of infants in in the present study were introduced to complementary food after four months of age and before 5.5 months of age, which is in line with the Norwegian recommendations from 2016. Introduction before four months of age was seen for 5% of the infants, which is somewhat lower than the 7% in the latest national cross-sectional survey from 2013 [7]. The higher proportion of well-educated mothers in our study may explain the lower number of children introduced to solid foods before four months of age, as education is known to affect health through mechanisms such as health literacy and health behaviors [53]. However, a lower proportion of children in our study (14%) had not been introduced to solid food at 5.5 months of age compared to the latest national survey (24%). This could indicate a real change over time or it could reflect possible selection bias. There was a high proportion of first-time mothers in our sample, and first-time mothers have been shown to introduce solid food earlier than multiparous mothers [37].

Our findings are comparable to previous findings in Denmark and Sweden [14, 54]. Outside Scandinavia, a national representative cross-sectional study from the US found that 16% of the infants were introduced to solid food before four months, while 45% of were not introduced to solid food at six months of age [55]. An Irish prospective study found that 18% of the infants were given solid food before 17 weeks and 79% before six months of age [56]. In Australia, a cohort study found that 44% of infants had received solid food before 17 weeks and 93% of infants had received solid food before 26 weeks of age [13]. Except for the US study, this study is in line with other studies from developed countries finding that only a minor proportion of the infants are introduced to their first solid food at six months of age.

### Infant characteristics

In line with previous research [18, 57], the boys in our study tended to be introduced to solid foods earlier than girls. Being a girl was an independent predictor of later introduction of complementary food. A possible explanation may be that male infants consume more breast/formula milk, feed more frequently and wakes up more often at nights. This may lead to earlier weaning instead of supplementing with additional breast/formula milk [18, 58, 59].

Preterm infants are more likely to be to be early introduced to solid food [20]. Possible reasons may be that parents consider chronological age as opposed to corrected age, experience more stress related to infant growth or that preterm infants are more prone to irritability and unexplained crying resulting in parents feeding to soothe. In our study we found no significant difference between the timing-of-introduction groups for whether the infant was born before or during/after gestational week 38. However, only 14% of the infants born before week 38 had a reported birthweight below 2500 g, of which only one infant below 2000 g (1920g). We have no further data regarding actual gestational age, but the reported birthweights indicate that the preterm infants in this sample are born moderate to late preterm (32 to 37 weeks). A previous study found that infants born more premature had the greatest odds of early introduction [20], this may explain our findings.

The association between age at introduction of complementary feeding and risk of later childhood overweight or obesity has been debated. Early introduction of complementary food have been associated with rapid or excessive weight gain [60], which in systematic reviews have been identified as a risk factor for later overweight [61, 62]. A large population-based Australian study including 3153 infants found that both early (before 4 months) and late (after 7 months) introduction of solid food was associated with increased odds of above normal BMI at 1 year of age, independent of infants´ breastfeeding status at 4 months [63]. However, a recent systematic review of systematic reviews found no consistent evidence of an association between early introduction of solid food and later overweight [64]. In our study, introduction of solid food before 5 months of age was associated with a greater weight gain. Infants in the two earliest timing-of-introduction groups had the highest zBMI-change values from 3–5 months, although the difference only reached significance for the group of infants introduced to solid food between 4 and 5 months of age. The difference in weight-for-age z-scores (WAZ) from 3–5 months was significantly higher in the group of infants introduced to solid food before 4 months. Rapid weight gain (RWG) is defined as an increase of > 0.67 in sex-specific WAZ score within a specific time period [65]. Although the criteria for RWG was not met, the weight development in the early introduction group nevertheless gives cause for concern. A recently published systematic review found strong associations between RWG during infancy and subsequent overweight/obesity risk, with higher odds of overweight/obesity with RWG from birth to 1 year than RWG from birth to two years [65].

Parents report that a major reason to introduce solid food is to improve infant sleeping patterns. Sleep duration of <12 h during infancy (age 6–24 months) is also a risk factor for overweight and adiposity in preschoolers [66]. We found no significant differences between the separate timing-of-introduction groups for infant hours of sleeping or frequent awakening, suggesting that sleeping difficulties was not associated with timing of complementary feeding in our sample.

Difficult infant temperament has been shown to influence parental feeding behavior [67, 68] and contribute to early introduction of complementary food [9, 37]. In Norway, infant temperament has also been associated with an obesogenic diet at 18 months of age [69]. In our study there was no significant difference in the fussy/difficult-temperament score of The Infant Characteristics Questionnaire between the groups. The infant mean-score in this sample was 2.5, slightly higher than the mean score found for infants at six months of age in the Norwegian Mother and Child Cohort study (2.3) [44]. Niegel et.al. described their mean score as a rather low score, indicating that the mothers tended to perceive their infant`s temperament as easy. This may also be the case in the present study and explain our findings.

### Breastfeeding and formula feeding

Infants introduced to solid food before four months of age, were less often exclusively breastfed the first month of age and were more often receiving only formula milk as milk type at three months of age. Receiving only breastmilk at three months of age, was an independent predictor of later introduction of complementary food. These findings are well line with previous research. A recently published national study from the US including 1482 children, reported that early introduction to complementary food (before four months of age) was more common among infants that were never breastfed or breastfed for less than four months [55].

### Maternal characteristics

Mothers introducing solid food before child age four months were characterized by being younger, not married, less educated, more frequently smoking and having more economical difficulties. While maternal smoking was an independent predictor for early introduction to solid food, this was not the case for use of snus. To our knowledge, this is the first study to find different associations for timing of complementary feeding and maternal smoking and use of snus, respectively. The use of snus has so far not been registered in the official birth registers, and there is a lack of information regarding how use of snus affects early feeding decisions like breastfeeding duration and timing of complementary feeding. Our findings may be related to a different social distribution regarding use of snus in the Norwegian population, where the social differences associated with use of snus are less than for smoking [70].

There was no significant difference in maternal BMI between the four timing-of-introduction groups. There was an observed tendency for a higher BMI in mothers who introduced solid food before child age 4 months, but the difference was not significant. The mean maternal BMI was relatively high (24.9) and can be explained by the mothers not having regained their pre-pregnancy weight.

We also found no association between maternal symptoms of anxiety and depression and the timing of introduction to solid foods. The mean SCL-8 score in our sample was 1.25, which is similar to the pre-pregnancy mean score found in previous Norwegian population-based studies [27, 33]. It`s estimated that 10–15% of Norwegian women, including those in the period after childbirth, have symptoms of depression [71]. The proportion of mothers with a SCL8 score above the recommended cut-off at 2.00 were ≤ 5.5% in all the timing-of-introduction groups. The self-selection of participants in this study may have led to an underrepresentation of mothers with mental difficulties and contributed to our findings.

Timing of complementary feeding should be sensitive to the nutritional needs of the infant. In our study we found that apart from the infant's gender, maternal characteristics appear to be decisive. A systematic review from 2009 found that the determinants with strongest evidence for early weaning were young maternal age, low maternal education, low socioeconomic status, absence or short duration of breastfeeding and maternal smoking–besides lack of information or advice from health care provider [21]. The associations between early feeding practices and infant gender, milk-feeding mode, maternal smoking and maternal socioeconomic status are previously seen in both Scandinavian countries [24, 37, 72, 73] and in developed countries outside Scandinavia [13, 56, 74], but some of these studies are based on study populations from more than 10 years back in time. Our study confirms previous findings and contributes to the existing body of research with updated knowledge, showing that young maternal age, low maternal education and maternal smoking still are important predictors for non-adherence to infant feeding recommendations in a western, developed country.

### Strengths and limitations

As this paper examines a cross-sectional sample of infant-mother dyads, causal conclusions cannot be drawn. The nature of the sample and the self-reported data also makes it difficult to generalize from our findings. The mothers in the present study volunteered for participation, which in epidemiological research is known to make a skewness towards a more well-educated population [75, 76]. This also applies to our study. A low variability in socioeconomic status biases results towards the null and may have resulted in an underestimation of socioeconomic characteristics. The small size of the early-introduction group may have resulted in observed differences not achieving statistical significance. Nevertheless, we chose to include this group because of its relevance for the group of mothers with lower socioeconomic status—a group of importance for this field. The use of self-reported data may reduce their reliability of our findings and may be particularly problematic for weight-related indicators. Both sleep-wake behaviors and temperament are known to be notably stable behavioral characteristics from infancy to toddlerhood [77]. Nevertheless, the retrospective collection of data may have affected the results regarding infant temperament and sleeping difficulties.

Despite these limitations, our study also has strengths. A comparatively large number of participants were included. Our results are well in line with previous research which supports the findings. To our knowledge, this is the first study that have found different associations between maternal smoking and use of snus and timing of complementary feeding. Furthermore, we have concurrently examined a wide range of both infant and maternal characteristics of importance for early infant feeding interactions with potential to inform public health policy.

## Conclusion

The majority of Norwegian infants are fed in accordance with national infant feeding recommendations. Predictors for early introduction to complementary food were breastfeeding status and maternal sociodemographic factors. Introduction of solid food before five months of age was associated with increased infant weight gain. Timing of introduction to complementary food was not, except for the infant's gender, associated with infant cues that may be markers of increased nutritional needs of the infant. Our results confirm that infants of younger, less educated and smoking mothers are still at higher risk of not being fed in compliance with the official infant feeding recommendations. Altogether, our findings emphasize the importance of targeting socioeconomically disadvantaged mothers for support on healthy feeding practices focusing on the infant's needs to prevent early onset of social inequalities in health with potential life-long duration. Larger population-based studies with a longitudinal design are needed to further explore the relationship between timing of complementary feeding, maternal socioeconomic status and infant growth trajectories.

## Supporting information

S1 Questionnaire Survey questionnaire in English. (PDF)

S2 Questionnaire Survey questionnaire in Norwegian. (PDF)

## References

[1] World Health Organization; Michael S. Kramer RK. The optimal duration of exclusive breastfeeding. A systematic review 2001. 10.1007/978-1-4757-4242-8_7 15384567

[2] World Health Organization & UNICEF. Global Strategy on Infant and Young Child Fedding2003.

[3] Kramer MS, Kakuma R. Optimal duration of exclusive breastfeeding. The Cochrane database of systematic reviews. 2012(8):Cd003517 doi: 10.1002/14651858.CD003517.pub2 22895934 10.1002/14651858.CD003517.pub2 PMC7154583

[4] Agostoni C, Braegger C, Decsi T, Kolacek S, Koletzko B, Michaelsen KF, et al Breast-feeding: A commentary by the ESPGHAN Committee on Nutrition. J Pediatr Gastroenterol Nutr. 2009;49(1):112–25. doi: 10.1097/MPG.0b013e31819f1e05 19502997 10.1097/MPG.0b013e31819f1e05

[5] Agostoni C, Decsi T, Fewtrell M, Goulet O, Kolacek S, Koletzko B, et al Complementary feeding: a commentary by the ESPGHAN Committee on Nutrition. J Pediatr Gastroenterol Nutr. 2008;46(1):99–110. doi: 10.1097/01.mpg.0000304464.60788.bd 18162844 10.1097/01.mpg.0000304464.60788.bd

[6] Caroli M, Mele RM, Tomaselli MA, Cammisa M, Longo F, Attolini E. Complementary feeding patterns in Europe with a special focus on Italy. Nutr Metab Cardiovasc Dis. 2012;22(10):813–8. doi: 10.1016/j.numecd.2012.07.007 22898449 10.1016/j.numecd.2012.07.007

[7] Norwegian Directorate of Health hhnpa-o-s-k-l-u-. Breast feeding and Infant Nutrition. 2014.

[8] Norwegian Directorate of health. The Norwegian dietary guidelines for infant nutrition. https://helsedirektoratet.no/retningslinjer/spedbarnsernering. 2017.

[9] Doub AE, Moding KJ, Stifter CA. Infant and maternal predictors of early life feeding decisions. The timing of solid food introduction. Appetite. 2015;92:261–8. doi: 10.1016/j.appet.2015.05.028 26025089 10.1016/j.appet.2015.05.028 PMC4499500

[10] Hodges EA, Johnson SL, Hughes SO, Hopkinson JM, Butte NF, Fisher JO. Development of the responsiveness to child feeding cues scale. Appetite. 2013;65:210–9. doi: 10.1016/j.appet.2013.02.010 23419965 10.1016/j.appet.2013.02.010 PMC3995412

[11] Pearce J, Taylor MA, Langley-Evans SC. Timing of the introduction of complementary feeding and risk of childhood obesity: a systematic review. Int J Obes (Lond). 2013;37(10):1295–306. 23736360 10.1038/ijo.2013.99

[12] Clayton HB, Li R, Perrine CG, Scanlon KS. Prevalence and reasons for introducing infants early to solid foods: variations by milk feeding type. Pediatrics. 2013;131(4):e1108–14. doi: 10.1542/peds.2012-2265 23530169 10.1542/peds.2012-2265 PMC3608486

[13] Scott JA, Binns CW, Graham KI, Oddy WH. Predictors of the early introduction of solid foods in infants: results of a cohort study. BMC Pediatr. 2009;9:60 doi: 10.1186/1471-2431-9-60 19772610 10.1186/1471-2431-9-60 PMC2754451

[14] Kronborg H, Foverskov E, Vaeth M. Breastfeeding and introduction of complementary food in Danish infants. Scandinavian journal of public health. 2015;43(2):138–45. doi: 10.1177/1403494814567171 25630521 10.1177/1403494814567171

[15] Smith HA, Becker GE. Early additional food and fluids for healthy breastfed full-term infants. The Cochrane database of systematic reviews. 2016;8:Cd006462. 10.1002/14651858.CD006462.pub4 PMC8588276 27574798

[16] Walsh A, Kearney L, Dennis N. Factors influencing first-time mothers' introduction of complementary foods: a qualitative exploration. BMC Public Health. 2015;15:939 doi: 10.1186/s12889-015-2250-z 26395331 10.1186/s12889-015-2250-z PMC4580114

[17] Harrison M, Brodribb W, Hepworth J. A qualitative systematic review of maternal infant feeding practices in transitioning from milk feeds to family foods. Maternal & child nutrition. 2017;13(2). 10.1111/mcn.12360 PMC6865989 27696658

[18] Brown A, Rowan H. Maternal and infant factors associated with reasons for introducing solid foods. Maternal & child nutrition. 2016;12(3):500–15. 25721759 10.1111/mcn.12166 PMC6860142

[19] McMeekin S, Jansen E, Mallan K, Nicholson J, Magarey A, Daniels L. Associations between infant temperament and early feeding practices. A cross-sectional study of Australian mother-infant dyads from the NOURISH randomised controlled trial. Appetite. 2013;60(1):239–45. doi: 10.1016/j.appet.2012.10.005 23079142 10.1016/j.appet.2012.10.005

[20] Braid S, Harvey EM, Bernstein J, Matoba N. Early introduction of complementary foods in preterm infants. J Pediatr Gastroenterol Nutr. 2015;60(6):811–8. doi: 10.1097/MPG.0000000000000695 25564809 10.1097/MPG.0000000000000695

[21] Wijndaele K, Lakshman R, Landsbaugh JR, Ong KK, Ogilvie D. Determinants of early weaning and use of unmodified cow's milk in infants: a systematic review. J Am Diet Assoc. 2009;109(12):2017–28. doi: 10.1016/j.jada.2009.09.003 19942019 10.1016/j.jada.2009.09.003

[22] Kristiansen AL, Lande B, Overby NC, Andersen LF. Factors associated with exclusive breast-feeding and breast-feeding in Norway. Public Health Nutr. 2010;13(12):2087–96. doi: 10.1017/S1368980010002156 20707948 10.1017/S1368980010002156

[23] Gudnadottir M, Gunnarsson BS, Thorsdottir I. Effects of sociodemographic factors on adherence to breastfeeding and other important infant dietary recommendations. Acta Paediatr. 2006;95(4):419–24. doi: 10.1080/0803520500434769 16720488 10.1080/0803520500434769

[24] Erkkola M, Salmenhaara M, Nwaru BI, Uusitalo L, Kronberg-Kippila C, Ahonen S, et al Sociodemographic determinants of early weaning: a Finnish birth cohort study in infants with human leucocyte antigen-conferred susceptibility to type 1 diabetes. Public Health Nutr. 2013;16(2):296–304. doi: 10.1017/S1368980012002595 22607723 10.1017/S1368980012002595 PMC10271657

[25] Andren Aronsson C, Uusitalo U, Vehik K, Yang J, Silvis K, Hummel S, et al Age at first introduction to complementary foods is associated with sociodemographic factors in children with increased genetic risk of developing type 1 diabetes. Maternal & child nutrition. 2015;11(4):803–14. 24034553 10.1111/mcn.12084 PMC4122645

[26] Norwegian Institute of Public Health. Health risk when using snus. 2014.

[27] Ystrom E, Niegel S, Klepp KI, Vollrath ME. The impact of maternal negative affectivity and general self-efficacy on breastfeeding: the Norwegian Mother and Child Cohort Study. J Pediatr. 2008;152(1):68–72. doi: 10.1016/j.jpeds.2007.06.005 18154903 10.1016/j.jpeds.2007.06.005

[28] Haycraft E, Farrow C, Blissett J. Maternal symptoms of depression are related to observations of controlling feeding practices in mothers of young children. J Fam Psychol. 2013;27(1):159–64. doi: 10.1037/a0031110 23421843 10.1037/a0031110

[29] Ystrom E, Niegel S, Vollrath ME. The impact of maternal negative affectivity on dietary patterns of 18-month-old children in the Norwegian Mother and Child Cohort Study. Maternal & child nutrition. 2009;5(3):234–42. 20572926 10.1111/j.1740-8709.2008.00177.x PMC6860799

[30] Hurley KM, Black MM, Papas MA, Caulfield LE. Maternal symptoms of stress, depression, and anxiety are related to nonresponsive feeding styles in a statewide sample of WIC participants. J Nutr. 2008;138(4):799–805. doi: 10.1093/jn/138.4.799 18356338 10.1093/jn/138.4.799 PMC3137941

[31] Ystrom E, Barker M, Vollrath ME. Impact of mothers' negative affectivity, parental locus of control and child-feeding practices on dietary patterns of 3-year-old children: the MoBa Cohort Study. Maternal & child nutrition. 2012;8(1):103–14. 20738782 10.1111/j.1740-8709.2010.00257.x PMC6860815

[32] Hurley KM, Black MM, Merry BC, Caulfield LE. Maternal mental health and infant dietary patterns in a statewide sample of Maryland WIC participants. Maternal & child nutrition. 2015;11(2):229–39. 10.1111/mcn.12004 PMC6860248 23167622

[33] Hampson SE, Tonstad S, Irgens LM, Meltzer HM, Vollrath ME. Mothers' negative affectivity during pregnancy and food choices for their infants. Int J Obes (Lond). 2010;34(2):327–31. 19918247 10.1038/ijo.2009.230 PMC2822132

[34] Gaffney KF, Kitsantas P, Brito A, Swamidoss CSS. Postpartum Depression, Infant Feeding Practices, and Infant Weight Gain at Six Months of Age. J Pediatr Health Care. 2014;28(1):43–50. doi: 10.1016/j.pedhc.2012.10.005 23266435 10.1016/j.pedhc.2012.10.005

[35] Winkvist A, Brantsæter AL, Brandhagen M, Haugen M, Meltzer HM, Lissner L. Maternal Prepregnant Body Mass Index and Gestational Weight Gain Are Associated with Initiation and Duration of Breastfeeding among Norwegian Mothers. J Nutr. 2015;145(6):1263–70. doi: 10.3945/jn.114.202507 25904732 10.3945/jn.114.202507 PMC4442110

[36] Baker JL, Michaelsen KF, Sorensen TI, Rasmussen KM. High prepregnant body mass index is associated with early termination of full and any breastfeeding in Danish women. Am J Clin Nutr. 2007;86(2):404–11. doi: 10.1093/ajcn/86.2.404 17684212 10.1093/ajcn/86.2.404

[37] Kronborg H, Foverskov E, Vaeth M. Predictors for early introduction of solid food among Danish mothers and infants: an observational study. BMC Pediatr. 2014;14:243 doi: 10.1186/1471-2431-14-243 25270266 10.1186/1471-2431-14-243 PMC4263048

[38] Helle C, Hillesund ER, Omholt ML, Overby NC. Early food for future health: a randomized controlled trial evaluating the effect of an eHealth intervention aiming to promote healthy food habits from early childhood. BMC Public Health. 2017;17(1):729 doi: 10.1186/s12889-017-4731-8 28931384 10.1186/s12889-017-4731-8 PMC5607575

[39] University of Agder. Early Food for Future Health 2016 [Available from: http://spedbarnsmat.no/.

[40] Norwegian Health Directorate. Spedkost 6 months: Nationwide diet survey among 6 months old children. 2008.

[41] Norwegian Institute of Public Health. The Norwegian Mother and Child Cohort Study (MoBa) 1998–2008 [Available from: https://www.fhi.no/en/studies/moba/.

[42] World Health Organization. Child growth standards 2018 [Available from: http://www.who.int/childgrowth/software/en/.

[43] Bates JE, Freeland CA, Lounsbury ML. Measurement of infant difficultness. Child Dev. 1979;50(3):794–803. 498854

[44] Niegel S, Ystrom E, Vollrath ME. Is difficult temperament related to overweight and rapid early weight gain in infants? A prospective cohort study. J Dev Behav Pediatr. 2007;28(6):462–6. doi: 10.1097/DBP.0b013e31811431e8 18091091 10.1097/DBP.0b013e31811431e8

[45] World Health Organozation. Exclusive breastfeeding http://www.who.int/nutrition/topics/exclusive_breastfeeding/en/ 2017

[46] World Health Organization. BMI classification 2017 [Available from: http://apps.who.int/bmi/index.jsp?introPage=intro_3.html.

[47] Holseter C, Dalen JD, Krokstad S, Eikemo TA. Self-rated health and mortality in different occupational classes and income groups in Nord-Trondelag County, Norway. Tidsskr Nor Laegeforen. 2015;135(5):434–8. doi: 10.4045/tidsskr.13.0788 25761028 10.4045/tidsskr.13.0788

[48] Derogatis LR, Lipman RS, Covi L. SCL-90: an outpatient psychiatric rating scale—preliminary report. Psychopharmacol Bull. 1973;9(1):13–28. 4682398

[49] Tambs K, Røysamb E. Selection of questions to short-form versions of original psychometric instruments in MoBa. Norsk Epidemiologi. 2014;24(1–2):195–201.

[50] Kvalevaag AL, Ramchandani PG, Hove O, Eberhard-Gran M, Assmus J, Havik OE, et al Parents' Prenatal Mental Health and Emotional, Behavioral and Social Development in Their Children. Child Psychiatry Hum Dev. 2015;46(6):874–83. doi: 10.1007/s10578-014-0527-6 25504529 10.1007/s10578-014-0527-6

[51] Fink P, Orbol E, Hansen MS, Sondergaard L, De Jonge P. Detecting mental disorders in general hospitals by the SCL-8 scale. J Psychosom Res. 2004;56(3):371–5. doi: 10.1016/S0022-3999(03)00071-0 15046976 10.1016/S0022-3999(03)00071-0

[52] Norwegian Institute of Public Health. medical birth register http://statistikkbank.fhi.no/mfr/ 2017 [

[53] Cohen AK, Syme SL. Education: a missed opportunity for public health intervention. Am J Public Health. 2013;103(6):997–1001. doi: 10.2105/AJPH.2012.300993 23597373 10.2105/AJPH.2012.300993 PMC3698749

[54] Brekke HK, Ludvigsson JF, van Odijk J, Ludvigsson J. Breastfeeding and introduction of solid foods in Swedish infants: the All Babies in Southeast Sweden study. Br J Nutr. 2005;94(3):377–82. 16176608 10.1079/bjn20051499

[55] Barrera CM, Hamner HC, Perrine CG, Scanlon KS. Timing of Introduction of Complementary Foods to US Infants, National Health and Nutrition Examination Survey 2009–2014. Journal of the Academy of Nutrition and Dietetics. 2018;118(3):464–70. doi: 10.1016/j.jand.2017.10.020 29307590 10.1016/j.jand.2017.10.020 PMC5828913

[56] O'Donovan SM, Murray DM, Hourihane JO, Kenny LC, Irvine AD, Kiely M. Adherence with early infant feeding and complementary feeding guidelines in the Cork BASELINE Birth Cohort Study. Public Health Nutr. 2015;18(15):2864–73. doi: 10.1017/S136898001500018X 25690944 10.1017/S136898001500018X PMC10271636

[57] Grote V, Schiess SA, Closa-Monasterolo R, Escribano J, Giovannini M, Scaglioni S, et al The introduction of solid food and growth in the first 2 y of life in formula-fed children: analysis of data from a European cohort study. Am J Clin Nutr. 2011;94(6 Suppl):1785s–93s. doi: 10.3945/ajcn.110.000810 21918213 10.3945/ajcn.110.000810

[58] da Costa TH, Haisma H, Wells JC, Mander AP, Whitehead RG, Bluck LJ. How much human milk do infants consume? Data from 12 countries using a standardized stable isotope methodology. J Nutr. 2010;140(12):2227–32. doi: 10.3945/jn.110.123489 20980653 10.3945/jn.110.123489 PMC3592484

[59] Schrempft S, van Jaarsveld CH, Fisher A, Wardle J. Family and infant characteristics associated with timing of core and non-core food introduction in early childhood. Eur J Clin Nutr. 2013;67(6):652–7. doi: 10.1038/ejcn.2013.63 23486509 10.1038/ejcn.2013.63 PMC3674911

[60] Adair L. How could complementary feeding patterns affect the susceptibility to NCD later in life? Nutr Metab Carbiovasc Dis 2012 p. 765–9. 10.1016/j.numecd.2012.03.011 22901844

[61] Ong KK, Loos RJ. Rapid infancy weight gain and subsequent obesity: systematic reviews and hopeful suggestions. Acta Paediatr. 2006;95(8):904–8. doi: 10.1080/08035250600719754 16882560 10.1080/08035250600719754

[62] Weng SF, Redsell SA, Swift JA, Yang M, Glazebrook CP. Systematic review and meta-analyses of risk factors for childhood overweight identifiable during infancy. Arch Dis Child. 2012;97(12):1019–26. doi: 10.1136/archdischild-2012-302263 23109090 10.1136/archdischild-2012-302263 PMC3512440

[63] Sun C, Foskey RJ, Allen KJ, Dharmage SC, Koplin JJ, Ponsonby AL, et al The Impact of Timing of Introduction of Solids on Infant Body Mass Index. J Pediatr. 2016;179:104–10.e1. doi: 10.1016/j.jpeds.2016.08.064 27663213 10.1016/j.jpeds.2016.08.064

[64] Patro-Golab B, Zalewski BM, Kolodziej M, Kouwenhoven S, Poston L, Godfrey KM, et al Nutritional interventions or exposures in infants and children aged up to 3 years and their effects on subsequent risk of overweight, obesity and body fat: a systematic review of systematic reviews. Obes Rev. 2016;17(12):1245–57. doi: 10.1111/obr.12476 27749991 10.1111/obr.12476 PMC5325317

[65] Zheng M, Lamb KE, Grimes C, Laws R, Bolton K, Ong KK, et al Rapid weight gain during infancy and subsequent adiposity: a systematic review and meta-analysis of evidence. Obes Rev. 2018;19(3):321–32. doi: 10.1111/obr.12632 29052309 10.1111/obr.12632 PMC6203317

[66] Taveras EM, Rifas-Shiman SL, Oken E, Gunderson EP, Gillman MW. Short sleep duration in infancy and risk of childhood overweight. Arch Pediatr Adolesc Med. 2008;162(4):305–11. doi: 10.1001/archpedi.162.4.305 18391138 10.1001/archpedi.162.4.305 PMC2650815

[67] McNally J, Hugh-Jones S, Caton S, Vereijken C, Weenen H, Hetherington M. Communicating hunger and satiation in the first 2 years of life: a systematic review. Maternal & child nutrition. 2016;12(2):205–28. 26620159 10.1111/mcn.12230 PMC4991302

[68] Bergmeier H, Skouteris H, Horwood S, Hooley M, Richardson B. Associations between child temperament, maternal feeding practices and child body mass index during the preschool years: a systematic review of the literature. Obes Rev. 2014;15(1):9–18. doi: 10.1111/obr.12066 23957249 10.1111/obr.12066

[69] Vollrath ME, Tonstad S, Rothbart MK, Hampson SE. Infant temperament is associated with potentially obesogenic diet at 18 months. Int J Pediatr Obes. 2011;6(2–2):e408–14. doi: 10.3109/17477166.2010.518240 20854098 10.3109/17477166.2010.518240 PMC3128685

[70] Norwegian Institute of Public Health. Smoking and use of snus in Norway 2017 [Available from: https://www.fhi.no/ml/royking/.

[71] Norwegian Institute of Public Health; Dalgard OS. Levekårsundersøkelsen 2006 https://www.fhi.no/publ/eldre/levekarsundersokelsen-2005.-psykisk/. 2005.

[72] Lande B, Andersen LF, Baerug A, Trygg KU, Lund-Larsen K, Veierod MB, et al Infant feeding practices and associated factors in the first six months of life: the Norwegian infant nutrition survey. Acta Paediatr. 2003;92(2):152–61. 12710639 10.1111/j.1651-2227.2003.tb00519.x

[73] Klingberg S, Ludvigsson J, Brekke HK. Introduction of complementary foods in Sweden and impact of maternal education on feeding practices. Public Health Nutr. 2017;20(6):1054–62. doi: 10.1017/S1368980016003104 27917749 10.1017/S1368980016003104 PMC10261380

[74] Castro PD, Kearney J, Layte R. A study of early complementary feeding determinants in the Republic of Ireland based on a cross-sectional analysis of the Growing Up in Ireland infant cohort. Public Health Nutr. 2015;18(2):292–302. doi: 10.1017/S1368980014000329 24642376 10.1017/S1368980014000329 PMC10271045

[75] Nilsen RM, Vollset SE, Gjessing HK, Skjaerven R, Melve KK, Schreuder P, et al Self-selection and bias in a large prospective pregnancy cohort in Norway. Paediatr Perinat Epidemiol. 2009;23(6):597–608. doi: 10.1111/j.1365-3016.2009.01062.x 19840297 10.1111/j.1365-3016.2009.01062.x

[76] Nilsen RM, Suren P, Gunnes N, Alsaker ER, Bresnahan M, Hirtz D, et al Analysis of self-selection bias in a population-based cohort study of autism spectrum disorders. Paediatr Perinat Epidemiol. 2013;27(6):553–63. doi: 10.1111/ppe.12077 23919580 10.1111/ppe.12077 PMC3851582

[77] Hayes MJ, McCoy SK, Fukumizu M, Wellman JD, Dipietro JA. Temperament and Sleep-Wake Behaviors from Infancy to Toddlerhood. Infant and child development. 2011;20(5):495–508. doi: 10.1002/icd.720 22003317 10.1002/icd.720 PMC3190304

