# Supplementary material for: Timing of complementary feeding and associations with maternal and infant characteristics: A Norwegian cross-sectional study
Source: PLoS One. 2018 Jun 27;13(6):e0199455. doi: 10.1371/journal.pone.0199455 (PMC6021099; doi:10.1371/journal.pone.0199455)
Supplement: S1 Questionnaire — (PDF) [file pone.0199455.s001.pdf]

Thank you very much for taking part in the Baby e-Food Study!

This is the first of two questionnaires we will ask you to fill out. The second will be sent to you when your child becomes one year.

It will take about 30 minutes to fill out the questionnaire. It's easiest to fill out the form on a computer. You navigate in the form by pressing the arrows / "next button" at the bottom of the page.

After some initial questions, the questionnaire is two-fold:
The first part of the questionnaire is about the child participating in the survey, which is now approx. 6 months old. The second part is about you who is the mother or father of the child.

Good luck!

Date of completion of form
Must be written DD.MM:YYYY
Eg 22.12.2015____

How did you get information about this study?

(1) ❑ Through the child health service
(2) ❑ Through social media/facebook
(3) ❑ Through both child health service and social media/Facebook
(4) ❑ None, describe _____

What is your relationship with the child participating in the survey?

(1) ❑ Mother
(2) ❑ Father
(3) ❑ None, describe _____

Do you live with the father/mother of the child participating in the survey?

(1) ❑ Yes
(2) ❑ No

Does the child's other parent want to respond to the survey by completing a separate questionnaire?

(1) ❑ yes

(2) ❑ no

Enter his / her email address in the box below.

An email with a new link to the survey will be sent to the specified email address within the next few days.

_____

Repeat e-mail:

_____

Now follows the first part of the questionnaire with questions about the child participating in the survey.
There are questions regarding growth and development, the child's eating habits and the child's temperament and sleep. Finally in this section there are some questions about child rearing.
You will need the child's health card for information on weight and height.

What is the child's date of birth?
Must be written DD.MM:YYYY

____

What is the child's gender?

(1) ❑ girl

(2) ❑ boy

Was the child born in/after pregnancy week 38?

(1) ❑ yes
(2) ❑ no

What was the child's birth weight and length?

Birth weight in grams _____

Birth length in cm _____

Based on the child's health card from the child health center, enter the date of examination, weight and length in the questions below:

Examination at ap. 6 weeks of age:

Date of investigation _____

weight in grams _____

length in cm _____

Examination at ap. 3 months of age:

Date of investigation _____

weight in grams _____

length in cm _____

Examination at ap. 5 months of age:

Date of investigation _____

weight in grams _____

length in cm _____

All in all; How will you characterize your child's physical health?

(1) ❑ Very good
(5) ❑ Good
(6) ❑ Poor
(7) ❑ Very poor

How often does the child wake up at night nowadays?

(1) ❑ 3 or more times each night
(2) ❑ 1-2 times each night
(3) ❑ Sometimes during the week
(4) ❑ Rarely or never

How many hours do the child usually sleep per 24h?

(1) ❑ Less than 8 hours
(2) ❑ 8-10 hours
(3) ❑ 11-12 hours
(4) ❑ 13-14 hours
(5) ❑ More than 14 hours

How often is the child outdoors?

(1) ❑ Seldom
(2) ❑ Often, but less than an hour daily
(3) ❑ 1-3 hours daily
(4) ❑ More than 3 hours daily

Where is the baby taken care of during the day now?

(1) ❑ Home with mother / father
(2) ❑ Home with nanny / trainee
(3) ❑ At nanny
(4) ❑ In family run kindergarten
(5) ❑ In Kindergarden
(6) ❑ Other, describe _____

Now follow questions about the baby's diet and eating habits:

What did the child drink first week?
You can select multiple options

(1) ❑ Breast milk
(2) ❑ Water
(3) ❑ Sugar Water
(4) ❑ Infant formula
(5) ❑ Other, describe _____
(6) ❑ Do not know / do not remember

What kind of foods and drinks have been given to the child for the first 6 months?

Tic each month the child has received the current drink or food

	**0 months**	**1 months**	**2 months**	**3 months**	**4 months**	**5 months**	**6 months**
Breast milk	(1) ❑	(10) ❑	(11) ❑	(12) ❑	(13) ❑	(14) ❑	(15) ❑
Infant formula, all types	(1) ❑	(10) ❑	(11) ❑	(12) ❑	(13) ❑	(14) ❑	(15) ❑
Water	(1) ❑	(10) ❑	(11) ❑	(12) ❑	(13) ❑	(14) ❑	(15) ❑
Saft / juice	(1) ❑	(10) ❑	(11) ❑	(12) ❑	(13) ❑	(14) ❑	(15) ❑
Baby porridge	(1) ❑	(10) ❑	(11) ❑	(12) ❑	(13) ❑	(14) ❑	(15) ❑
Dinner	(1) ❑	(10) ❑	(11) ❑	(12) ❑	(13) ❑	(14) ❑	(15) ❑
Fruit / berries	(1) ❑	(10) ❑	(11) ❑	(12) ❑	(13) ❑	(14) ❑	(15) ❑

How often does the child have the following drink nowadays?

	**Never / seldom**	**1-3 times per week**	**4-6 times per week**	**1 time a day**	**2 times a day**	**3 times a day**	**4 times a day**	**5 or more times a day**
Breast milk	(1) ❑	(2) ❑	(3) ❑	(4) ❑	(5) ❑	(6) ❑	(7) ❑	(8) ❑
Infant formula, all types	(1) ❑	(2) ❑	(3) ❑	(4) ❑	(5) ❑	(6) ❑	(7) ❑	(8) ❑
Milk, all types	(1) ❑	(2) ❑	(3) ❑	(4) ❑	(5) ❑	(6) ❑	(7) ❑	(8) ❑
Sour milk (e.g. yoghurt)	(1) ❑	(2) ❑	(3) ❑	(4) ❑	(5) ❑	(6) ❑	(7) ❑	(8) ❑
Chocolate milk, all types	(1) ❑	(2) ❑	(3) ❑	(4) ❑	(5) ❑	(6) ❑	(7) ❑	(8) ❑

	Never / seldom	1-3 times per week	4-6 times per week	1 time a day	2 times a day	3 times a day	4 times a day	5 or more times a day
Boiled water	(1) ❑	(2) ❑	(3) ❑	(4) ❑	(5) ❑	(6) ❑	(7) ❑	(8) ❑
Tap water	(1) ❑	(2) ❑	(3) ❑	(4) ❑	(5) ❑	(6) ❑	(7) ❑	(8) ❑
Water bought in bottle	(1) ❑	(2) ❑	(3) ❑	(4) ❑	(5) ❑	(6) ❑	(7) ❑	(8) ❑

How often does the child have the following drink nowadays?

	Never / seldom	1-3 times per week	4-6 times per week	1 time a day	2 times a day	3 times a day	4 times a day	5 or more times a day
Baby limonade from bottle	(1) ❑	(2) ❑	(3) ❑	(4) ❑	(5) ❑	(6) ❑	(7) ❑	(8) ❑
Other limonade, with sugar	(1) ❑	(2) ❑	(3) ❑	(4) ❑	(5) ❑	(6) ❑	(7) ❑	(8) ❑
Limonade, sweetener	(1) ❑	(2) ❑	(3) ❑	(4) ❑	(5) ❑	(6) ❑	(7) ❑	(8) ❑
Juice	(1) ❑	(2) ❑	(3) ❑	(4) ❑	(5) ❑	(6) ❑	(7) ❑	(8) ❑
Mineral water with sugar	(1) ❑	(2) ❑	(3) ❑	(4) ❑	(5) ❑	(6) ❑	(7) ❑	(8) ❑
Mineral water, sweetener	(1) ❑	(2) ❑	(3) ❑	(4) ❑	(5) ❑	(6) ❑	(7) ❑	(8) ❑

Is it you who usually gives the baby food?

(1) ❑ Yes
(2) ❑ No
(3) ❑ Share alike

How often does the child eat the following food nowadays?

	How often						
	Never / seldom	1-3 /week	4-6 / week	1 /day	2 / day	3 / day	≥ 4 /day
Industrial-made porridge, all types	(1) ❑	(2) ❑	(3) ❑	(4) ❑	(5) ❑	(6) ❑	(7) ❑
Homemade porridge of whole-grain flour or oatmeal	(1) ❑	(2) ❑	(3) ❑	(4) ❑	(5) ❑	(6) ❑	(7) ❑
Homemade porridge of millet	(1) ❑	(2) ❑	(3) ❑	(4) ❑	(5) ❑	(6) ❑	(7) ❑

	How often						
	Never / seldom	1-3 /week	4-6 / week	1 /day	2 / day	3 / day	≥ 4 /day
Homemade porridge of white flour, rusk, semule, rice, or corn	(1) ❑	(2) ❑	(3) ❑	(4) ❑	(5) ❑	(6) ❑	(7) ❑

How often does the child eat the following food nowadays?

	How often?						
	Never / seldom	1-3 /week	4-6 / week	1 /day	2 / day	3 / day	≥ 4 /day
Industrial-made dinner with vegetables	(1) ❑	(2) ❑	(3) ❑	(4) ❑	(5) ❑	(6) ❑	(7) ❑
Industrial-made dinner on with vegetables and meat / poultry	(1) ❑	(2) ❑	(3) ❑	(4) ❑	(5) ❑	(6) ❑	(7) ❑
Industrial-made dinner with vegetables and fish	(1) ❑	(2) ❑	(3) ❑	(4) ❑	(5) ❑	(6) ❑	(7) ❑

How often does the child eat the following food nowadays?

	How often?						
	Never / seldom	1-3 /week	4-6 / week	1 /day	2 / day	3 / day	≥ 4 /day
Homemade dinner, potato or vegetable mash	(1) ❑	(2) ❑	(3) ❑	(4) ❑	(5) ❑	(6) ❑	(7) ❑
Homemade dinner with meat / poultry and vegetables	(1) ❑	(2) ❑	(3) ❑	(4) ❑	(5) ❑	(6) ❑	(7) ❑
Homemade dinner with fish and vegetables	(1) ❑	(2) ❑	(3) ❑	(4) ❑	(5) ❑	(6) ❑	(7) ❑
Other home made dinner	(1) ❑	(2) ❑	(3) ❑	(4) ❑	(5) ❑	(6) ❑	(7) ❑

How often does the child eat the following food nowadays?

	How often?						
	Never / seldom	1-3 /week	4-6 / week	1 /day	2 / day	3 / day	≥ 4 /day
Industry-made fruit / berries mash	(1) ❑	(2) ❑	(3) ❑	(4) ❑	(5) ❑	(6) ❑	(7) ❑

	How often?						
	**Never / seldom**	**1-3 /week**	**4-6 / week**	**1 /day**	**2 / day**	**3 / day**	**≥ 4 /day**
Homemade fruit / berries mash	(1) ❑	(2) ❑	(3) ❑	(4) ❑	(5) ❑	(6) ❑	(7) ❑

How often does the child eat the following food nowadays?

	How often?						
	**Never / seldom**	**1-3 /week**	**4-6 / week**	**1 /day**	**2 / day**	**3 / day**	**≥ 4 /day**
Bread	(1) ❑	(2) ❑	(3) ❑	(4) ❑	(5) ❑	(6) ❑	(7) ❑
Yogurt	(1) ❑	(2) ❑	(3) ❑	(4) ❑	(5) ❑	(6) ❑	(7) ❑
Ice	(1) ❑	(2) ❑	(3) ❑	(4) ❑	(5) ❑	(6) ❑	(7) ❑
Biscuits / cakes	(1) ❑	(2) ❑	(3) ❑	(4) ❑	(5) ❑	(6) ❑	(7) ❑
snacks	(1) ❑	(2) ❑	(3) ❑	(4) ❑	(5) ❑	(6) ❑	(7) ❑

How old was the child when she / he was introduced to the following food:

	**Not tasted**	**0 mos**	**1 mos**	**2 mos**	**3 mos**	**4 mos**	**5 mos**	**6 mos**
Vegetable mash, homemade	(1) ❑	(4) ❑	(5) ❑	(6) ❑	(7) ❑	(8) ❑	(9) ❑	(10) ❑
Vegetable mash, industrial made	(1) ❑	(4) ❑	(5) ❑	(6) ❑	(7) ❑	(8) ❑	(9) ❑	(10) ❑
Fruit mash/smoothie, homemade	(1) ❑	(4) ❑	(5) ❑	(6) ❑	(7) ❑	(8) ❑	(9) ❑	(10) ❑
Fruit mash/smoothie, industrial made	(1) ❑	(4) ❑	(5) ❑	(6) ❑	(7) ❑	(8) ❑	(9) ❑	(10) ❑

How old was the child when she / he was introduced to the following food:

	**Not tasted**	**0 mos**	**1 mos**	**2 mos**	**3 mos**	**4 mos**	**5 mos**	**6 mos**
Porridge, home made	(1) ❑	(4) ❑	(5) ❑	(6) ❑	(7) ❑	(8) ❑	(9) ❑	(10) ❑
Porridge, industrial made	(1) ❑	(4) ❑	(5) ❑	(6) ❑	(7) ❑	(8) ❑	(9) ❑	(10) ❑
Dinner with meat / poultry / fish, homemade	(1) ❑	(4) ❑	(5) ❑	(6) ❑	(7) ❑	(8) ❑	(9) ❑	(10) ❑
Dinner with meat / poultry / fish, industrial made	(1) ❑	(4) ❑	(5) ❑	(6) ❑	(7) ❑	(8) ❑	(9) ❑	(10) ❑

	Not tasted	0 mos	1 mos	2 mos	3 mos	4 mos	5 mos	6 mos
Bread	(1) ❑	(4) ❑	(5) ❑	(6) ❑	(7) ❑	(8) ❑	(9) ❑	(10) ❑

Does the child get cod liver oi, vitamins, iron or other supplements?

(1) ❑ Yes

(2) ❑ No

Specify type of dietary supplement, amount and frequency.

	Number of T-spoons?					How often?		
	**Not applica ble**	**1**	**2**	**3**	**≥4**	**Not applicable**	**daily**	**Now and then**
Cod liver oil	(1) ❑	(2) ❑	(5) ❑	(4) ❑	(3) ❑	(1) ❑	(2) ❑	(3) ❑
Fluent multivitamin supplement	(1) ❑	(2) ❑	(5) ❑	(4) ❑	(3) ❑	(1) ❑	(2) ❑	(3) ❑
Other	(1) ❑	(2) ❑	(5) ❑	(4) ❑	(3) ❑	(1) ❑	(2) ❑	(3) ❑

Hvor gammelt var barnet da det begynte med kosttilskuddet?

	Age in months							
	**Not applicabl e**	**0**	**1**	**2**	**3**	**4**	**5**	**6**
Cod liver oil	(2) ❑	(10) ❑	(9) ❑	(5) ❑	(4) ❑	(3) ❑	(6) ❑	(7) ❑
Fluent multivitamin supplement	(2) ❑	(10) ❑	(9) ❑	(5) ❑	(4) ❑	(3) ❑	(6) ❑	(7) ❑
Other	(2) ❑	(10) ❑	(9) ❑	(5) ❑	(4) ❑	(3) ❑	(6) ❑	(7) ❑

Now follow some questions about the child's behavior in meals and how you experience this. Think about the way it use to be when answering the questions.

How would you describe your child's eating behavior during a typical day meal?

	Never	Rarely	Sometimes	Often	Always
My baby seems contented while feeding	(1) ❑	(2) ❑	(3) ❑	(4) ❑	(5) ❑
My baby frequently wants more milk than I provide	(1) ❑	(2) ❑	(3) ❑	(4) ❑	(5) ❑
My baby loves milk	(1) ❑	(2) ❑	(3) ❑	(4) ❑	(5) ❑
My baby has a big appetite	(1) ❑	(2) ❑	(3) ❑	(4) ❑	(5) ❑
My baby finishes feeding quickly	(1) ❑	(2) ❑	(3) ❑	(4) ❑	(5) ❑
My baby becomes distressed while feeding	(1) ❑	(2) ❑	(3) ❑	(4) ❑	(5) ❑

How would you describe your child's eating behavior during a typical day meal?

	Never	Rarely	Sometimes	Often	Always
My baby gets full up easily	(1) ❑	(2) ❑	(3) ❑	(4) ❑	(5) ❑
If allowed to, my baby would take too much milk	(1) ❑	(2) ❑	(3) ❑	(4) ❑	(5) ❑
My baby takes more than 30 minutes to finish feeding	(1) ❑	(2) ❑	(3) ❑	(4) ❑	(5) ❑
My baby gets full before taking all the milk I think he/she should have	(1) ❑	(2) ❑	(3) ❑	(4) ❑	(5) ❑
My baby feeds slowly	(1) ❑	(2) ❑	(3) ❑	(4) ❑	(5) ❑
Even when my baby has just eaten well he/she is happy to feed again if offered	(1) ❑	(2) ❑	(3) ❑	(4) ❑	(5) ❑

How would you describe your child's eating behavior during a typical day meal?

	Never	Rarely	Sometimes	Often	Always
My baby finds it difficult to manage a complete feed	(1) ❑	(2) ❑	(3) ❑	(4) ❑	(5) ❑
My baby is always demanding a feed	(1) ❑	(2) ❑	(3) ❑	(4) ❑	(5) ❑

	Never	Rarely	Sometimes	Often	Always
My baby sucks more and more slowly during the course of a feed	(1) ❑	(2) ❑	(3) ❑	(4) ❑	(5) ❑
If given the chance, my baby would always be feeding	(1) ❑	(2) ❑	(3) ❑	(4) ❑	(5) ❑
My baby enjoys feeding time	(1) ❑	(2) ❑	(3) ❑	(4) ❑	(5) ❑
My baby can easily take a feed within 30 minutes of the last one	(1) ❑	(2) ❑	(3) ❑	(4) ❑	(5) ❑

How often do you do or experience the following?

	Never	Seldom	Sometimes	Often	Always
Do you let s-/he eat whenever s-/he want to?	(1) ❑	(2) ❑	(3) ❑	(4) ❑	(5) ❑
Do you worry that s-/he does not eat enough?	(1) ❑	(2) ❑	(3) ❑	(4) ❑	(5) ❑
Do you only allow her/him to eat at set times?	(1) ❑	(2) ❑	(3) ❑	(4) ❑	(5) ❑
When s-/he get fuzzy, is feeding her/him the first thing you would do?	(1) ❑	(2) ❑	(3) ❑	(4) ❑	(5) ❑
Do you worry that s-/he is eating to much?	(1) ❑	(2) ❑	(3) ❑	(4) ❑	(5) ❑
Is it a struggle to get her/him to eat?	(1) ❑	(2) ❑	(3) ❑	(4) ❑	(5) ❑
Do you get upset if s-/he eats too much?	(1) ❑	(2) ❑	(3) ❑	(4) ❑	(5) ❑
To make sure s-/he doesn`t get fuzzy, do you feed her/him even if you don`t think s-/he is hungry?	(1) ❑	(2) ❑	(3) ❑	(4) ❑	(5) ❑

To what extent do you agree or disagree with the statements below?

	Disagree a lot	Disagree a little	Neither-nor	Agree a little	Agree a lot
If I don`t encourage her/him to eat, then s-/he will not eat enough.	(1) ❑	(2) ❑	(3) ❑	(4) ❑	(5) ❑
Feeding her/him is the best way to stop her/his fussiness	(1) ❑	(2) ❑	(3) ❑	(4) ❑	(5) ❑
I know when s-/he is hungry	(1) ❑	(2) ❑	(3) ❑	(4) ❑	(5) ❑
I am worried that s-/he will become overweight	(1) ❑	(2) ❑	(3) ❑	(4) ❑	(5) ❑
I know when s-/he is full	(1) ❑	(2) ❑	(3) ❑	(4) ❑	(5) ❑
My child knows when s-/he is hungry	(1) ❑	(2) ❑	(3) ❑	(4) ❑	(5) ❑
I am worried that s-/he will become underweight	(1) ❑	(2) ❑	(3) ❑	(4) ❑	(5) ❑
My child knows when s-/he is full	(1) ❑	(2) ❑	(3) ❑	(4) ❑	(5) ❑

How confident do you feel about the following:

	Not confident at all	Somewhat inconfident	Neither-nor	Somewhat confident	Very confident
Give baby healthy food	(1) ❑	(2) ❑	(3) ❑	(4) ❑	(5) ❑
Can get baby to eat enough	(1) ❑	(2) ❑	(3) ❑	(4) ❑	(5) ❑
Can get baby to try vegetables	(1) ❑	(2) ❑	(3) ❑	(4) ❑	(5) ❑
Give baby right amount of food	(1) ❑	(2) ❑	(3) ❑	(4) ❑	(5) ❑
Can get baby to taste new food	(1) ❑	(2) ❑	(3) ❑	(4) ❑	(5) ❑

Here follows some questions about the child's general behavior.

Tick to what extent you agree or disagree with the following statements about the child's mood and temperament.

	Highly disagree	Disagree	Slightly disagree	Neither-nor	Slightly agree	Agree	Highly agree
The baby cries a lot	(1) ❑	(2) ❑	(3) ❑	(4) ❑	(5) ❑	(6) ❑	(7) ❑
The child is usually easy to calm when she / he cries	(1) ❑	(2) ❑	(3) ❑	(4) ❑	(5) ❑	(6) ❑	(7) ❑
The child gets easy upset and starts to cry	(1) ❑	(2) ❑	(3) ❑	(4) ❑	(5) ❑	(6) ❑	(7) ❑
When the child is crying, she / he usually cries loudly and vigorously	(1) ❑	(2) ❑	(3) ❑	(4) ❑	(5) ❑	(6) ❑	(7) ❑
She / he is easy to handle	(1) ❑	(2) ❑	(3) ❑	(4) ❑	(5) ❑	(6) ❑	(7) ❑

Tick to what extent you agree or disagree with the following statements about the child's mood and temperament.

	Highly disagree	Disagree	Slightly disagree	Neither-nor	Slightly agree	Agree	Highly agree
The child requires a lot of attention	(1) ❑	(2) ❑	(3) ❑	(4) ❑	(5) ❑	(6) ❑	(7) ❑
When the child is left to itself, he / she usually plays well with him / herself	(1) ❑	(2) ❑	(3) ❑	(4) ❑	(5) ❑	(6) ❑	(7) ❑
The child is so demanding that he / she would represent a significant problem for most parents	(1) ❑	(2) ❑	(3) ❑	(4) ❑	(5) ❑	(6) ❑	(7) ❑
The child smiles and laughs often	(1) ❑	(2) ❑	(3) ❑	(4) ❑	(5) ❑	(6) ❑	(7) ❑
The child is easy to put to sleep, and fell asleep quickly	(1) ❑	(2) ❑	(3) ❑	(4) ❑	(5) ❑	(6) ❑	(7) ❑

Finally in this section there are some questions about child rearing:

How do you agree with the following statements?

	Highly agree	Slightly agree	Neither-nor	Slightly disagree	Highly disagree
You can spoil a baby	(1) ❑	(2) ❑	(3) ❑	(4) ❑	(5) ❑
My baby needs to learn the difference between what is right and wrong	(1) ❑	(2) ❑	(3) ❑	(4) ❑	(5) ❑
Babies should be encouraged to entertain themselves	(1) ❑	(2) ❑	(3) ❑	(4) ❑	(5) ❑
It is very important that my b. meets their developemental milestones on time	(1) ❑	(2) ❑	(3) ❑	(4) ❑	(5) ❑
I have a strict day to day routine for my baby	(1) ❑	(2) ❑	(3) ❑	(4) ❑	(5) ❑
I do lots of organized activities with my baby	(1) ❑	(2) ❑	(3) ❑	(4) ❑	(5) ❑
It is never too young to start disciplining a child	(1) ❑	(2) ❑	(3) ❑	(4) ❑	(5) ❑
Sometimes my baby cries to try and manipulate me	(1) ❑	(2) ❑	(3) ❑	(4) ❑	(5) ❑

How do you agree with the following statements?

	Highly agree	Slightly agree	Neither-nor	Slightly disagree	Highly disagree
Babies need a routine	(1) ❑	(2) ❑	(3) ❑	(4) ❑	(5) ❑
I regularly ask other people advice about my baby`s behaviour	(1) ❑	(2) ❑	(3) ❑	(4) ❑	(5) ❑
I make sure I play, read or sing with my baby very regularly	(1) ❑	(2) ❑	(3) ❑	(4) ❑	(5) ❑
I make sure I put my baby down regularly	(1) ❑	(2) ❑	(3) ❑	(4) ❑	(5) ❑
My baby sometimes do things that are naughty	(1) ❑	(2) ❑	(3) ❑	(4) ❑	(5) ❑
I think people not using a routineare making a rod for their own back	(1) ❑	(2) ❑	(3) ❑	(4) ❑	(5) ❑
I worry a lot about my baby	(1) ❑	(2) ❑	(3) ❑	(4) ❑	(5) ❑

	Highly agree	Slightly agree	Neither-nor	Slightly disagree	Highly disagree
Cuddling babies makes them too dependent	(1) ❑	(2) ❑	(3) ❑	(4) ❑	(5) ❑

How do you agree with the following statements?

	Highly agree	Slightly agree	Neither-nor	Slightly disagree	Highly disagree
My baby sets their own routine	(1) ❑	(2) ❑	(3) ❑	(4) ❑	(5) ❑
I encourage my baby to develop their skills such as walking or talking	(1) ❑	(2) ❑	(3) ❑	(4) ❑	(5) ❑
I often check baby books to see if my baby is on target	(1) ❑	(2) ❑	(3) ❑	(4) ❑	(5) ❑
I generally like to keep my baby as close as possible to me	(1) ❑	(2) ❑	(3) ❑	(4) ❑	(5) ❑
Everyone is happiest when the baby is in a routine	(1) ❑	(2) ❑	(3) ❑	(4) ❑	(5) ❑
I regularly seek advice from my helath visitor or GP about my baby	(1) ❑	(2) ❑	(3) ❑	(4) ❑	(5) ❑
Babies under 1 year do not need disipline	(1) ❑	(2) ❑	(3) ❑	(4) ❑	(5) ❑
A routine makes a baby calm and secure	(1) ❑	(2) ❑	(3) ❑	(4) ❑	(5) ❑
Babies need a lot of parental input such as play, reading and actvities	(1) ❑	(2) ❑	(3) ❑	(4) ❑	(5) ❑

Now follows the second part of the questionnaire with questions about yourself.
The questions deal with background information, eating habits and your experience of your own health.

What is your date of birth?
Must be written DD. MM.YYYY. Eg 22.12.2015_______________________________________

What civil status do you have now?

(1) ❑ Married

(2) ❑ Cohabitant

(3) ❑ Single

(4) ❑ Divorced

(5) ❑ Widow / widower

(6) ❑ Other, describe _____

How many people are there in your household?

Number of adults _____

Number of children _____

Age of children not participating in the survey
answer in years and put a comma between each child; eg. 3, 5_____

Are you, ev. mother of the child participating in the survey, pregnant now?

(1) ❑ Yes

(2) ❑ No

Do you or your child's other parent have a mother tongue other than Norwegian?

(1) ❑ Yes

(2) ❑ No

Which native language, describe

(1) ❑ Mother _____

(2) ❑ Dad _____

Does one of the child's grandparents have a mother tongue other than Norwegian?

(1) ❑ Yes

(2) ❑ No

Which native language, describe

(1) ❑ maternal grandmother _____

(2) ❑ maternal grandfather _____

(3) ❑ paternal grandmother _____

(4) ❑ paternal grandfather _____

What education do you have?
Choose the highest completed education

(1) ❑ Less than 9/10 years primary school

(2) ❑ Primary school

(3) ❑ High school

(4) ❑ Highschool, vocational

(5) ❑ University / college up to 4 years

(6) ❑ University / college more than 4 years

(8) ❑ Other education

What is your main activity?
Ev. What was your main activity before you became pregnant?

(1) ❑ Working full time

(2) ❑ Working part-time

(3) ❑ Homemakers

(4) ❑ Sick leave

(5) ❑ Leave

(6) ❑ Disability benefits

(7) ❑ During rehabilitation

(8) ❑ Student

(9) ❑ Unemployed

(10) ❑ Other

Which county do you live in?

(1) ❑ Akershus

(2) ❑ Aust-Agder

(3) ❑ Buskerud

(4) ❑ Finnmark

(5) ❑ Hedmark

(6) ❑ Hordaland

(7) ❑ Møre og Romsdal

(8) ❑ Nord-Trøndelag
(9) ❑ Nordland
(10) ❑ Oppland
(11) ❑ Oslo
(12) ❑ Rogaland
(13) ❑ Sogn og Fjordane
(14) ❑ Sør-Trøndelag
(15) ❑ Telemark
(16) ❑ Troms
(18) ❑ Vest-Agder
(17) ❑ Vestfold
(19) ❑ Østfold

How many residents live in your nearest town?

(1) ❑ 0-4999
(2) ❑ 5000-14999
(3) ❑ 15000-49999
(4) ❑ More than 50000

Do you own your own home?

(1) ❑ Yes
(2) ❑ No

Are you able to pay an unforeseen expence of 3000 NOK, e.g. for a repair or a dental bill?

(1) ❑ Yes
(2) ❑ No
(3) ❑ Don't know

Has it occurred during the past six months that you / you have had difficulty coping with expenses for food, transport, rent and the like?

(1) ❑ No, never
(2) ❑ Yes, seldom
(3) ❑ Yes, sometimes
(4) ❑ Yes, often

Now follow some questions about living habits and lifestyle:

How tall are you?
Answer in centimeters

______

How much do you weigh now?
Answer in kg

Are you trying to lose weight?

(1) ☐ No, my weight is appropriate
(2) ☐ No, I need to gain weight
(3) ☐ No, but I need to loose weight
(4) ☐ Yes

Do you smoke?

(1) ☐ No, never smoked regularly
(2) ☐ No, has quitted
(3) ☐ Yes, but not daily
(4) ☐ Yes, daily

Do you use snus (powdered tobacco)?

(1) ☐ No, never used snus regularly
(2) ☐ No, has quitted
(3) ☐ Yes, but not daily
(4) ☐ Yes, daily

How many times a week are you so physically active that you get short-breathed or sweat?
Duration at least 30 min. per time

(1) ☐ Never
(2) ☐ Less than 1 time per week

(3) ❑ 1 time per week
(4) ❑ 2 times per week
(5) ❑ 3-4 times per week
(6) ❑ 5 times per week

In your spare time; how much time do you spend on a TV, PC / tablet or smartphone?

(1) ❑ Less than an hour daily
(2) ❑ Between 1 and 2 hours daily
(3) ❑ Between 2 and 4 hours daily
(4) ❑ Between 4 and 6 hours daily
(5) ❑ More than 6 hours daily

Now follow some questions about diet and eating habits.
We ask about your eating habits as they usually are. We realize that the diet varies from day to day, so try to give an "average" of your eating habits the way they have been in the past year.

How would you describe your own diet?

(1) ❑ I have a regular varied diet
(2) ❑ I do not eat fish
(3) ❑ I do not eat meat
(4) ❑ I am a vegetarian
(5) ❑ I follow a special diet, describe: ______
(6) ❑ None of the descriptions fit

How many times do you usually eat the following meals during a week?

	**Never / rarely**	**1 / w**	**2 / w**	**3 / w**	**4 / w**	**5 / w**	**6 / w**	**Each day**
Breakfast	(1) ❑	(2) ❑	(3) ❑	(4) ❑	(5) ❑	(6) ❑	(7) ❑	(8) ❑
Lunch	(1) ❑	(2) ❑	(3) ❑	(4) ❑	(5) ❑	(6) ❑	(7) ❑	(8) ❑
A snack before dinner	(1) ❑	(2) ❑	(3) ❑	(4) ❑	(5) ❑	(6) ❑	(7) ❑	(8) ❑
Dinner	(1) ❑	(2) ❑	(3) ❑	(4) ❑	(5) ❑	(6) ❑	(7) ❑	(8) ❑

	Never / rarely	1 / w	2 / w	3 / w	4 / w	5 / w	6 / w	Each day
A snack after dinner	(1) ❑	(2) ❑	(3) ❑	(4) ❑	(5) ❑	(6) ❑	(7) ❑	(8) ❑
Supper	(1) ❑	(2) ❑	(3) ❑	(4) ❑	(5) ❑	(6) ❑	(7) ❑	(8) ❑
Other meals	(1) ❑	(2) ❑	(3) ❑	(4) ❑	(5) ❑	(6) ❑	(7) ❑	(8) ❑

Do you have the main responsibility for cooking at home?

(1) ❑ Yes
(2) ❑ No
(3) ❑ The responsibility is shared

How often do you do the following?

	Never	<1 / w	1 / w	2 / w	3 / w	4 / w	5 / w	6 / w	Daily
Cuts up vegetables	(1) ❑	(2) ❑	(3) ❑	(4) ❑	(5) ❑	(6) ❑	(7) ❑	(8) ❑	(9) ❑
Cuts up fruit	(1) ❑	(2) ❑	(3) ❑	(4) ❑	(5) ❑	(6) ❑	(7) ❑	(8) ❑	(9) ❑
Preparing dinner with raw materials	(1) ❑	(2) ❑	(3) ❑	(4) ❑	(5) ❑	(6) ❑	(7) ❑	(8) ❑	(9) ❑

Do you eat fast food more than once a week?

(1) ❑ Yes
(2) ❑ No

How much do you usually drink of the following drinks?

An unit is the same as a glass or a cup

	Never / rarely	1-3 unit / month	1-3 unit / week	4-6 units / week	1-3 unit / day	4-6 units / day	≥7 units / day
Milk	(1) ❑	(2) ❑	(3) ❑	(4) ❑	(5) ❑	(6) ❑	(7) ❑
Fat reduced milk	(1) ❑	(2) ❑	(3) ❑	(4) ❑	(5) ❑	(6) ❑	(7) ❑
Orange juice / fruit juice	(1) ❑	(2) ❑	(3) ❑	(4) ❑	(5) ❑	(6) ❑	(7) ❑
Limonade with sugar	(1) ❑	(2) ❑	(3) ❑	(4) ❑	(5) ❑	(6) ❑	(7) ❑
Limonade with sweetener	(1) ❑	(2) ❑	(3) ❑	(4) ❑	(5) ❑	(6) ❑	(7) ❑

How much do you usually drink of the following drinks?

An unit is the same as a glass or a cup

	**Never / rarely**	**1-3 unit / month**	**1-3 unit / week**	**4-6 units / week**	**1-3 unit / day**	**4-6 units / day**	**≥7 units / day**
Mineralwater with sugar	(1) ❑	(2) ❑	(3) ❑	(4) ❑	(5) ❑	(6) ❑	(7) ❑
Mineralwater with sweetener	(1) ❑	(2) ❑	(3) ❑	(4) ❑	(5) ❑	(6) ❑	(7) ❑
Coffee	(1) ❑	(2) ❑	(3) ❑	(4) ❑	(5) ❑	(6) ❑	(7) ❑
Tea	(1) ❑	(2) ❑	(3) ❑	(4) ❑	(5) ❑	(6) ❑	(7) ❑
Beer	(1) ❑	(2) ❑	(3) ❑	(4) ❑	(5) ❑	(6) ❑	(7) ❑
Wine	(1) ❑	(2) ❑	(3) ❑	(4) ❑	(5) ❑	(6) ❑	(7) ❑

How often do you eat the following foods?

	**Never / rarely**	**1-3 / month**	**1-3 / week**	**4-6 / week**	**1 / day**	**2 / day**	**3 / day**	**≥4 / day**
Boiled potatoes	(1) ❑	(2) ❑	(4) ❑	(5) ❑	(6) ❑	(7) ❑	(8) ❑	(9) ❑
French fries	(1) ❑	(2) ❑	(4) ❑	(5) ❑	(6) ❑	(7) ❑	(8) ❑	(9) ❑
Rice	(1) ❑	(2) ❑	(4) ❑	(5) ❑	(6) ❑	(7) ❑	(8) ❑	(9) ❑
pasta	(1) ❑	(2) ❑	(4) ❑	(5) ❑	(6) ❑	(7) ❑	(8) ❑	(9) ❑
wholegrain Pasta	(1) ❑	(2) ❑	(4) ❑	(5) ❑	(6) ❑	(7) ❑	(8) ❑	(9) ❑
Raw vegetables / salads	(1) ❑	(2) ❑	(4) ❑	(5) ❑	(6) ❑	(7) ❑	(8) ❑	(9) ❑
Boiled vegetables	(1) ❑	(2) ❑	(4) ❑	(5) ❑	(6) ❑	(7) ❑	(8) ❑	(9) ❑

How often do you eat the following foods?

	**Never / rarely**	**1-3 / month**	**1-3 / week**	**4-6 / week**	**1 / day**	**2 / day**	**3 / day**	**≥4 / day**
Fish (cooked or fried)	(1) ❑	(2) ❑	(4) ❑	(5) ❑	(6) ❑	(7) ❑	(8) ❑	(9) ❑
Fishburgers / fish balls	(1) ❑	(2) ❑	(4) ❑	(5) ❑	(6) ❑	(7) ❑	(8) ❑	(9) ❑
Minced meat	(1) ❑	(2) ❑	(4) ❑	(5) ❑	(6) ❑	(7) ❑	(8) ❑	(9) ❑
Pure meat	(1) ❑	(2) ❑	(4) ❑	(5) ❑	(6) ❑	(7) ❑	(8) ❑	(9) ❑
Chicken / turkey	(1) ❑	(2) ❑	(4) ❑	(5) ❑	(6) ❑	(7) ❑	(8) ❑	(9) ❑
Pizza	(1) ❑	(2) ❑	(4) ❑	(5) ❑	(6) ❑	(7) ❑	(8) ❑	(9) ❑

	Never / rarely	1-3 / month	1-3 / week	4-6 / week	1 / day	2 / day	3 / day	≥4 / day
Sausages / hamburger	(1) ❑	(2) ❑	(4) ❑	(5) ❑	(6) ❑	(7) ❑	(8) ❑	(9) ❑

How often do you eat the following foods?

	Never / rarely	1-3 / month	1-3 / week	4-6 / week	1 / day	2 / day	3 / day	≥4 / day
Fruit	(1) ❑	(2) ❑	(4) ❑	(5) ❑	(6) ❑	(7) ❑	(8) ❑	(9) ❑
Berries	(1) ❑	(2) ❑	(4) ❑	(5) ❑	(6) ❑	(7) ❑	(8) ❑	(9) ❑
Wholegrain bread	(1) ❑	(2) ❑	(4) ❑	(5) ❑	(6) ❑	(7) ❑	(8) ❑	(9) ❑
White bread	(1) ❑	(2) ❑	(4) ❑	(5) ❑	(6) ❑	(7) ❑	(8) ❑	(9) ❑
Cakes, biscuits	(1) ❑	(2) ❑	(4) ❑	(5) ❑	(6) ❑	(7) ❑	(8) ❑	(9) ❑
Desserts, ice cream	(1) ❑	(2) ❑	(4) ❑	(5) ❑	(6) ❑	(7) ❑	(8) ❑	(9) ❑
Sweets, candy	(1) ❑	(2) ❑	(4) ❑	(5) ❑	(6) ❑	(7) ❑	(8) ❑	(9) ❑
Chocolate	(1) ❑	(2) ❑	(4) ❑	(5) ❑	(6) ❑	(7) ❑	(8) ❑	(9) ❑
Potato chips	(1) ❑	(2) ❑	(4) ❑	(5) ❑	(6) ❑	(7) ❑	(8) ❑	(9) ❑
Peanuts	(1) ❑	(2) ❑	(4) ❑	(5) ❑	(6) ❑	(7) ❑	(8) ❑	(9) ❑

Do you use any kind of dietary supplement?

	Yes	No
Vitamin supplements	(1) ❑	(2) ❑
Cod liver oil	(1) ❑	(2) ❑

How do you agree with the following?

	Higly disagree	Moderat disagree	Slightly disagree	Neither-nor	Slightly agree	Moderat agree	Highly agree
I constantly taste new and different foods	(1) ❑	(5) ❑	(7) ❑	(6) ❑	(8) ❑	(9) ❑	(4) ❑
I do not trust new foods	(1) ❑	(5) ❑	(7) ❑	(6) ❑	(8) ❑	(9) ❑	(4) ❑
If I don’t know what a food is, I won`t try it	(1) ❑	(5) ❑	(7) ❑	(6) ❑	(8) ❑	(9) ❑	(4) ❑

	Higly disagree	Moderat disagree	Slightly disagree	Neither-nor	Slightly agree	Moderat agree	Highly agree
I like foods from different countrie	(1) ❑	(5) ❑	(7) ❑	(6) ❑	(8) ❑	(9) ❑	(4) ❑
Ethnic food looks weird to me	(1) ❑	(5) ❑	(7) ❑	(6) ❑	(8) ❑	(9) ❑	(4) ❑
At dinner parties, I will try new food	(1) ❑	(5) ❑	(7) ❑	(6) ❑	(8) ❑	(9) ❑	(4) ❑
I am afraid to eat things I have never had before	(1) ❑	(5) ❑	(7) ❑	(6) ❑	(8) ❑	(9) ❑	(4) ❑
I am very particular about the foods I eat	(1) ❑	(5) ❑	(7) ❑	(6) ❑	(8) ❑	(9) ❑	(4) ❑
I will eat almost anything	(1) ❑	(5) ❑	(7) ❑	(6) ❑	(8) ❑	(9) ❑	(4) ❑
I like to try ethnic restaurants	(1) ❑	(5) ❑	(7) ❑	(6) ❑	(8) ❑	(9) ❑	(4) ❑

Finally follows some questions about self-perceived physical and mental health:

All in all; How will you characterize your physical health?

(1) ❑ Very good
(5) ❑ Good
(6) ❑ Bad
(7) ❑ Very bad

To what extent does your health limit your everyday tasks?

(1) ❑ Largely
(2) ❑ To some degree
(3) ❑ To a small degree
(4) ❑ Not at all

Have you been bothered with any of the following in the last two weeks?

	Not at all	A little bit	Quite a bit	Extremely
Feeling fearful	(1) ❑	(2) ❑	(3) ❑	(4) ❑
Nervousness or shakiness inside	(1) ❑	(2) ❑	(3) ❑	(4) ❑

	Not at all	A little bit	Quite a bit	Extremely
Feeling hopeless about the future	(1) ❑	(2) ❑	(3) ❑	(4) ❑
Feeling blue	(1) ❑	(2) ❑	(3) ❑	(4) ❑
Worrying too much about thing	(1) ❑	(2) ❑	(3) ❑	(4) ❑
Feeling everything is an effort	(1) ❑	(2) ❑	(3) ❑	(4) ❑
Feeling tense or keyed up	(1) ❑	(2) ❑	(3) ❑	(4) ❑
Suddenly scared for no reason	(1) ❑	(2) ❑	(3) ❑	(4) ❑

How often do you experience the following in your daily life?

	Rarely / never	Hardly ever	Sometimes	Often	Very often
Feel glad about something	(1) ❑	(2) ❑	(3) ❑	(4) ❑	(5) ❑
Feel happy	(1) ❑	(2) ❑	(3) ❑	(4) ❑	(5) ❑
Feel joyful, like everything is going your way, everything is rosy	(1) ❑	(2) ❑	(3) ❑	(4) ❑	(5) ❑
Feel like screaming at somebody or banging on something	(1) ❑	(2) ❑	(3) ❑	(4) ❑	(5) ❑
Feel angry, irritated, annoyed	(1) ❑	(2) ❑	(3) ❑	(4) ❑	(5) ❑
Feel mad at somebody	(1) ❑	(2) ❑	(3) ❑	(4) ❑	(5) ❑

How true are these statements for you?

	Not at all true	Hardly true	Moderatly true	Exactly true
I can always manage to solve difficult problems if I try hard enough	(1) ❑	(2) ❑	(3) ❑	(4) ❑
If someone opposes me, I can find the means and ways to get what I want.	(1) ❑	(2) ❑	(3) ❑	(4) ❑
I am confident that I could deal efficiently with unexpected events.	(1) ❑	(2) ❑	(3) ❑	(4) ❑

	Not at all true	Hardly true	Moderatly true	Exactly true
I can remain calm when facing difficulties because I can rely on my coping abilities.	(1) ❑	(2) ❑	(3) ❑	(4) ❑
If I am in trouble, I can usually think of a solution.	(1) ❑	(2) ❑	(3) ❑	(4) ❑

Then you have completed filling out the form.
You submit it by pressing the button below.

Thank you!
