# Supplementary material for: Timing of complementary feeding and associations with maternal and infant characteristics: A Norwegian cross-sectional study
Source: PLoS One. 2018 Jun 27;13(6):e0199455. doi: 10.1371/journal.pone.0199455 (PMC6021099; doi:10.1371/journal.pone.0199455)
Supplement: S2 Questionnaire — (PDF) [file pone.0199455.s002.pdf]

Tusen takk for at du vil delta i barnE-mat studien!

Dette er det første av to spørreskjema vi vil be deg fylle ut. Det andre får du tilsendt når barnet ditt er blitt ett år.

Det vil ta ca. 30 minutter å fylle ut spørreskjemaet. Det er enklest å fylle ut skjemaet ved en dataskjerm. Du navigerer i skjemaet ved å trykke på pilene/"neste-knappen" nederst på siden. Det kan være lurt å fylle ut hele skjemaet på en gang, finn gjerne et tidspunkt der du kan sitte uforstyrret.

Etter noen innledende spørsmål er spørreskjemaet er todelt:
Første del av spørreskjemaet er om barnet som deltar i undersøkelsen, og som nå er ca. 6 måneder gammelt. Andre del er om deg som er mor eller far til barnet.

Lykke til!

Dato for utfylling av skjema
Må skrives dag.måned.år. Feks 22.12.2015

____

Hvordan fikk du informasjon om denne studien?

(1) ❑ Gjennom helsestasjonen
(2) ❑ Gjennom sosiale medier/facebook
(3) ❑ Gjennom både helsestasjon og sosiale medier
(4) ❑ Ingen av delene, beskriv _____

Hva er din relasjon til barnet som deltar i undersøkelsen?

(1) ❑ Jeg er mor
(2) ❑ Jeg er far
(3) ❑ Jeg er ingen av delene, beskriv _____

Bor du sammen med far/mor til barnet som deltar i undersøkelsen?

(1) ❑ Ja
(2) ❑ Nei

Ønsker barnets andre forelder å svare på undersøkelsen ved å fylle ut et eget spørreskjema?

(1) ❑ ja

(2) ❑ nei

Skriv inn hans/hennes epostadresse i feltet under.
En epost med ny lenke til undersøkelsen vil bli sendt til oppgitt epostadresse i løpet av de nærmeste dagene.

______

Gjenta epostadresse:

______

Nå følger den første delen av spørreskjemaet med spørsmål om barnet som deltar i undersøkelsen.
Spørsmålene omhandler vekst og utvikling, barnets spisevaner og barnets væremåte. Til slutt i denne delen er det noen spørsmål om barneoppdragelse.
Du vil trenge barnets helsekort for opplysninger om vekt og høyde.

Hva er barnets fødselsdato?
Må skrives dag.måned.år. Feks 22.12.2015

____

Hva er barnets kjønn?

(1) ❑ jente

(2) ❑ gutt

Ble barnet født etter svangerskapsuke 38?

(1) ☐ ja
(2) ☐ nei

Hva var barnets fødselsvekt og lengde?

Fødselsvekt i gram _____

Fødselslengde i cm _____

Ta utgangspunkt i barnets helsekort fra helsestasjonen og fyll inn dato for undersøkelse, vekt og lengde i spørsmålene under:

Undersøkelse ved ca 6 ukers alder:

Dato for undersøkelse _____

Vekt i gram _____

Lengde i cm _____

Undersøkelse ved ca 3 måneders alder:

Dato for undersøkelse _____

Vekt i gram _____

Lengde i cm _____

Undersøkelse ved 5-6 måneders alder:

Dato for undersøkelse _____

Vekt i gram _____

Lengde i cm _____

Alt i alt; hvordan vil du karakterisere ditt barns fysiske helse?

(1) ❑ Meget god

(5) ❑ God

(6) ❑ Dårlig

(7) ❑ Svært dårlig

Hvor ofte hender det at barnet våkner om natten nå for tiden?

(1) ❑ 3 eller flere ganger hver natt

(2) ❑ 1-2 ganger hver natt

(3) ❑ Noen ganger i uken

(4) ❑ Sjelden eller aldri

Hvor mange timer sover barnet vanligvis tilsammen per døgn?

(1) ❑ Mindre enn 8 timer

(2) ❑ 8-10 timer

(3) ❑ 11-12 timer

(4) ❑ 13-14 timer

(5) ❑ Mer enn 14 timer

Hvor ofte er barnet utendørs?

(1) ❑ Sjelden

(2) ❑ Ofte, men mindre enn en time daglig

(3) ❑ 1-3 timer daglig

(4) ❑ Mer enn 3 timer daglig

Hvor passes barnet som deltar i undersøkelsen på dagtid nå?

(1) ❑ Hjemme med mor/far

(2) ❑ Hjemme med dagmamma/praktikant

(3) ❑ Hos dagmamma

(4) ❑ I familiebarnehage

(5) ❑ I barnehage

(6) ❑ Annet sted, beskriv _____

Nå følger spørsmål om barnets kost og matvaner:

Hva fikk barnet å drikke første leveuke?
Du kan sette flere kryss

(1) ❑ Morsmelk
(2) ❑ Vann
(3) ❑ Sukkervann
(4) ❑ Morsmelkerstatning
(5) ❑ Annet, beskriv _____
(6) ❑ Vet ikke / husker ikke

Hva slags mat og drikke har banet fått de første 6 levemånedene?
Huk av for hver måned barnet har fått den aktuelle drikke

	0 mnd.	1 mnd.	2 mnd.	3 mnd.	4 mnd.	5 mnd.	6 mnd.
Morsmelk	(1) ❑	(10) ❑	(11) ❑	(12) ❑	(13) ❑	(14) ❑	(15) ❑
Morsmelkerstatning, alle typer	(1) ❑	(10) ❑	(11) ❑	(12) ❑	(13) ❑	(14) ❑	(15) ❑
Vann	(1) ❑	(10) ❑	(11) ❑	(12) ❑	(13) ❑	(14) ❑	(15) ❑
Saft/juice	(1) ❑	(10) ❑	(11) ❑	(12) ❑	(13) ❑	(14) ❑	(15) ❑
Babygrøt	(1) ❑	(10) ❑	(11) ❑	(12) ❑	(13) ❑	(14) ❑	(15) ❑
Middag	(1) ❑	(10) ❑	(11) ❑	(12) ❑	(13) ❑	(14) ❑	(15) ❑
Frukt/bær	(1) ❑	(10) ❑	(11) ❑	(12) ❑	(13) ❑	(14) ❑	(15) ❑

Hvor ofte får barnet følgende å drikke nå for tiden?

	Aldri/sjelden	1-3 ganger per uke	4-6 ganger per uke	1 gang per døgn	2 ganger per døgn	3 ganger per døgn	4 ganger per døgn	5 eller flere ganger per døgn
Morsmelk	(1) ❑	(2) ❑	(3) ❑	(4) ❑	(5) ❑	(6) ❑	(7) ❑	(8) ❑
Morsmelkerstatning, alle typer	(1) ❑	(2) ❑	(3) ❑	(4) ❑	(5) ❑	(6) ❑	(7) ❑	(8) ❑
Vanlig søt melk, alle typer	(1) ❑	(2) ❑	(3) ❑	(4) ❑	(5) ❑	(6) ❑	(7) ❑	(8) ❑

	Aldri/sjelden	1-3 ganger per uke	4-6 ganger per uke	1 gang per døgn	2 ganger per døgn	3 ganger per døgn	4 ganger per døgn	5 eller flere ganger per døgn
Surmelk (yoghurt, kulturmelk o.l.)	(1) ❑	(2) ❑	(3) ❑	(4) ❑	(5) ❑	(6) ❑	(7) ❑	(8) ❑
Sjokolademelk, alle typer	(1) ❑	(2) ❑	(3) ❑	(4) ❑	(5) ❑	(6) ❑	(7) ❑	(8) ❑
Kokt vann	(1) ❑	(2) ❑	(3) ❑	(4) ❑	(5) ❑	(6) ❑	(7) ❑	(8) ❑
Vann fra springen	(1) ❑	(2) ❑	(3) ❑	(4) ❑	(5) ❑	(6) ❑	(7) ❑	(8) ❑
Vann kjøpt på flaske	(1) ❑	(2) ❑	(3) ❑	(4) ❑	(5) ❑	(6) ❑	(7) ❑	(8) ❑

Hvor ofte får barnet følgende å drikke nå for tiden?

	Aldri/sjelden	1-3 ganger per uke	4-6 ganger per uke	1 gang per døgn	2 ganger per døgn	3 ganger per døgn	4 ganger per døgn	5 eller flere ganger per døgn
Barnesaft kjøpt på flaske	(1) ❑	(2) ❑	(3) ❑	(4) ❑	(5) ❑	(6) ❑	(7) ❑	(8) ❑
Annen saft, sukret	(1) ❑	(2) ❑	(3) ❑	(4) ❑	(5) ❑	(6) ❑	(7) ❑	(8) ❑
Saft, kunstig søtet	(1) ❑	(2) ❑	(3) ❑	(4) ❑	(5) ❑	(6) ❑	(7) ❑	(8) ❑
Juice	(1) ❑	(2) ❑	(3) ❑	(4) ❑	(5) ❑	(6) ❑	(7) ❑	(8) ❑
Brus, sukret	(1) ❑	(2) ❑	(3) ❑	(4) ❑	(5) ❑	(6) ❑	(7) ❑	(8) ❑
Lettbrus, kunstig søtet	(1) ❑	(2) ❑	(3) ❑	(4) ❑	(5) ❑	(6) ❑	(7) ❑	(8) ❑

Er det du som oftest gir barnet mat?

(1) ❑ Ja
(2) ❑ Nei
(3) ❑ Deler likt

Hvor ofte spiser barnet følgende mat nå for tiden?

	Hvor ofte?						
	Aldri/sjelden	1-3 g/u	4-6 g/u	1 g/døgn	2 g/døgn	3 g/døgn	4 el.flere g/døgn
Industrifremstilt grøt, alle typer	(1) ❑	(2) ❑	(3) ❑	(4) ❑	(5) ❑	(6) ❑	(7) ❑

Hvor ofte?

	Aldri/sjelden	1-3 g/u	4-6 g/u	1 g/døgn	2 g/døgn	3 g/døgn	4 el.flere g/døgn
Hjemmelaget grøt av grovt/sammalt mel eller havregryn/havremel	(1) ❑	(2) ❑	(3) ❑	(4) ❑	(5) ❑	(6) ❑	(7) ❑
Hjemmelaget grøt av hirse	(1) ❑	(2) ❑	(3) ❑	(4) ❑	(5) ❑	(6) ❑	(7) ❑
Hjemmelaget grøt av fint/hvitt mel, kavring, semule, ris, eller mais	(1) ❑	(2) ❑	(3) ❑	(4) ❑	(5) ❑	(6) ❑	(7) ❑

Hvor ofte spiser barnet følgende mat nå for tiden?

Hvor ofte?

	Aldri/sjelden	1-3 g/u	4-6 g/u	1 g/døgn	2 g/døgn	3 g/døgn	4 el.flere g/døgn
Industrifremstilt middag på glass med bare grønnsaker	(1) ❑	(2) ❑	(3) ❑	(4) ❑	(5) ❑	(6) ❑	(7) ❑
Industrifremstilt middag på glass med grønnsaker og kjøtt/fjærkre	(1) ❑	(2) ❑	(3) ❑	(4) ❑	(5) ❑	(6) ❑	(7) ❑
Industrifremstilt middag på glass med grønnsaker og fisk	(1) ❑	(2) ❑	(3) ❑	(4) ❑	(5) ❑	(6) ❑	(7) ❑

Hvor ofte spiser barnet følgende mat nå for tiden?

Hvor ofte?

	Aldri/sjelden	1-3 g/u	4-6 g/u	1 g/døgn	2 g/døgn	3 g/døgn	4 el.flere g/døgn
Hjemmelaget middag, bare potet- eller grønnsaksmos	(1) ❑	(2) ❑	(3) ❑	(4) ❑	(5) ❑	(6) ❑	(7) ❑
Hjemmelaget middag med kjøtt/fjærkre og grønnsaker	(1) ❑	(2) ❑	(3) ❑	(4) ❑	(5) ❑	(6) ❑	(7) ❑
Hjemmelaget middag med fisk og grønnsaker	(1) ❑	(2) ❑	(3) ❑	(4) ❑	(5) ❑	(6) ❑	(7) ❑
Annen hjemmelaget middag	(1) ❑	(2) ❑	(3) ❑	(4) ❑	(5) ❑	(6) ❑	(7) ❑

Hvor ofte spiser barnet følgende mat nå for tiden?

	Hvor ofte?						
	**Aldri/sjelden**	**1-3 g/u**	**4-6 g/u**	**1 g/døgn**	**2 g/døgn**	**3 g/døgn**	**4 el.flere g/døgn**
Industrifremstilt frukt- eller bærmos på glass eller i klemmepose (smoothie)	(1) ❑	(2) ❑	(3) ❑	(4) ❑	(5) ❑	(6) ❑	(7) ❑
Hjemmelaget frukt- eller bærmos	(1) ❑	(2) ❑	(3) ❑	(4) ❑	(5) ❑	(6) ❑	(7) ❑

Hvor ofte spiser barnet følgende mat nå for tiden?

	Hvor ofte?						
	**Aldri/sjelden**	**1-3 g/u**	**4-6 g/u**	**1 g/døgn**	**2 g/døgn**	**3 g/døgn**	**4 el.flere g/døgn**
Brød	(1) ❑	(2) ❑	(3) ❑	(4) ❑	(5) ❑	(6) ❑	(7) ❑
Yoghurt	(1) ❑	(2) ❑	(3) ❑	(4) ❑	(5) ❑	(6) ❑	(7) ❑
Is	(1) ❑	(2) ❑	(3) ❑	(4) ❑	(5) ❑	(6) ❑	(7) ❑
Kjeks/kaker	(1) ❑	(2) ❑	(3) ❑	(4) ❑	(5) ❑	(6) ❑	(7) ❑
Snacks	(1) ❑	(2) ❑	(3) ❑	(4) ❑	(5) ❑	(6) ❑	(7) ❑

Hvor gammelt var barnet da hun/han ble introdusert for følgende mat:

	**Ikke smakt**	**0 mnd.**	**1 mnd.**	**2 mnd.**	**3 mnd.**	**4 mnd.**	**5 mnd.**	**6 mnd.**
Grønnsaksmos, hjemmelaget	(1) ❑	(4) ❑	(5) ❑	(6) ❑	(7) ❑	(8) ❑	(9) ❑	(10) ❑
Grønnsaksmos, industrifremstilt	(1) ❑	(4) ❑	(5) ❑	(6) ❑	(7) ❑	(8) ❑	(9) ❑	(10) ❑
Fruktmos, hjemmelaget	(1) ❑	(4) ❑	(5) ❑	(6) ❑	(7) ❑	(8) ❑	(9) ❑	(10) ❑
Fruktmos (smoothie), industrifremstilt	(1) ❑	(4) ❑	(5) ❑	(6) ❑	(7) ❑	(8) ❑	(9) ❑	(10) ❑

Hvor gammelt var barnet da hun/han ble introdusert for følgende mat:

	Ikke smakt	0 mnd.	1 mnd.	2 mnd.	3 mnd.	4 mnd.	5 mnd.	6 mnd.
Grøt, hjemmelaget	(1) ❑	(4) ❑	(5) ❑	(6) ❑	(7) ❑	(8) ❑	(9) ❑	(10) ❑
Grøt, industrifremstilt (grøtpulver)	(1) ❑	(4) ❑	(5) ❑	(6) ❑	(7) ❑	(8) ❑	(9) ❑	(10) ❑
Middag med kjøtt/fjærkre/fisk, hjemmelaget	(1) ❑	(4) ❑	(5) ❑	(6) ❑	(7) ❑	(8) ❑	(9) ❑	(10) ❑
Middag med kjøtt/fjærkre/fisk, industrifremstilt	(1) ❑	(4) ❑	(5) ❑	(6) ❑	(7) ❑	(8) ❑	(9) ❑	(10) ❑
Brød	(1) ❑	(4) ❑	(5) ❑	(6) ❑	(7) ❑	(8) ❑	(9) ❑	(10) ❑

Får barnet tran, vitaminer, jern eller annet kosttilskudd?

(1) ❑ Ja

(2) ❑ Nei

Angi hva slags type kosttilskudd, mengde og hyppighet.

	Antall T-skjeer per gang?					Hvor ofte?		
	Ikke aktuelt	1	2	3	4 eller fler	Ikke aktuelt	daglig	av og til
Tran	(1) ❑	(2) ❑	(5) ❑	(4) ❑	(3) ❑	(1) ❑	(2) ❑	(3) ❑
Flytende multivitamintilskudd	(1) ❑	(2) ❑	(5) ❑	(4) ❑	(3) ❑	(1) ❑	(2) ❑	(3) ❑
Annet	(1) ❑	(2) ❑	(5) ❑	(4) ❑	(3) ❑	(1) ❑	(2) ❑	(3) ❑

Hvor gammelt var barnet da det begynte med kosttilskuddet?

	Alder i måneder							
	Ikke aktuelt	0	1	2	3	4	5	6
Tran	(2) ❑	(10) ❑	(9) ❑	(5) ❑	(4) ❑	(3) ❑	(6) ❑	(7) ❑
Flytende multivitamintilskudd	(2) ❑	(10) ❑	(9) ❑	(5) ❑	(4) ❑	(3) ❑	(6) ❑	(7) ❑
Annet kosttilskudd	(2) ❑	(10) ❑	(9) ❑	(5) ❑	(4) ❑	(3) ❑	(6) ❑	(7) ❑

Nå følger noen spørsmål om barnets atferd i måltider, og hvordan du opplever dette. Tenk på hvordan det vanligvis pleier å være når du svarer på spørsmålene.

Hvordan vil du beskrive ditt barns spiseatferd under et typisk dag-måltid?

	Aldri	Sjelden	Noen ganger	Ofte	Alltid
Barnet mitt virker tilfreds når det mates	(1) ❑	(2) ❑	(3) ❑	(4) ❑	(5) ❑
Barnet mitt vil ofte ha mer melk enn jeg gir henne/han	(1) ❑	(2) ❑	(3) ❑	(4) ❑	(5) ❑
Barnet mitt elsker melk	(1) ❑	(2) ❑	(3) ❑	(4) ❑	(5) ❑
Barnet mitt har stor appetitt	(1) ❑	(2) ❑	(3) ❑	(4) ❑	(5) ❑
Barnet mitt blir raskt ferdig med et måltid	(1) ❑	(2) ❑	(3) ❑	(4) ❑	(5) ❑
Barnet mitt blir urolig når det mates	(1) ❑	(2) ❑	(3) ❑	(4) ❑	(5) ❑

Hvordan vil du beskrive ditt barns spiseatferd under et typisk dag-måltid?

	Aldri	Sjelden	Noen ganger	Ofte	Alltid
Barnet mitt blir lett mett	(1) ❑	(2) ❑	(3) ❑	(4) ❑	(5) ❑
Hvis barnet mitt fikk muligheten, ville hun/han ha drukket for mye melk	(1) ❑	(2) ❑	(3) ❑	(4) ❑	(5) ❑
Barnet mitt bruker mer enn 30 minutter på å bli ferdig med et måltid	(1) ❑	(2) ❑	(3) ❑	(4) ❑	(5) ❑
Barnet mitt blir mett før hun/han drikker all melken jeg mener hun/han bør ha	(1) ❑	(2) ❑	(3) ❑	(4) ❑	(5) ❑
Barnet mitt spiser sakte	(1) ❑	(2) ❑	(3) ❑	(4) ❑	(5) ❑
Selv når barnet mitt nettopp har spist godt, er hun/han glad for å bli tilbudt mat på ny	(1) ❑	(2) ❑	(3) ❑	(4) ❑	(5) ❑

Hvordan vil du beskrive ditt barns spiseatferd under et typisk dag-måltid?

	Aldri	Sjelden	Noen ganger	Ofte	Alltid
Det er vanskelig for barnet mitt å gjennomføre et fullstendig måltid	(1) ❑	(2) ❑	(3) ❑	(4) ❑	(5) ❑
Barnet mitt krever alltid noe å spise	(1) ❑	(2) ❑	(3) ❑	(4) ❑	(5) ❑
Barnet mitt suger gradvis langsommere i løpet av et måltid	(1) ❑	(2) ❑	(3) ❑	(4) ❑	(5) ❑
Hvis barnet mitt fikk muligheten, ville hun/han alltid ha spist	(1) ❑	(2) ❑	(3) ❑	(4) ❑	(5) ❑
Barnet mitt koser seg i måltidssituasjonen	(1) ❑	(2) ❑	(3) ❑	(4) ❑	(5) ❑
Barnet mitt kan lett spise på ny, mindre enn 30 minutter etter at forrige måltid ble avsluttet	(1) ❑	(2) ❑	(3) ❑	(4) ❑	(5) ❑

Hvor ofte gjør eller opplever du følgende?

	Aldri	Sjelden	Noen ganger	Ofte	Alltid
Lar du barnet ditt få mat når det vil?	(1) ❑	(2) ❑	(3) ❑	(4) ❑	(5) ❑
Er du bekymret for at barnet ditt ikke spiser nok?	(1) ❑	(2) ❑	(3) ❑	(4) ❑	(5) ❑
Lar du barnet ditt bare få mat til bestemte tider?	(1) ❑	(2) ❑	(3) ❑	(4) ❑	(5) ❑
Når barnet ditt blir urolig, forsøker du først å gi henne/han noe å spise?	(1) ❑	(2) ❑	(3) ❑	(4) ❑	(5) ❑
Er du bekymret for at barnet ditt spiser for mye?	(1) ❑	(2) ❑	(3) ❑	(4) ❑	(5) ❑

	Aldri	Sjelden	Noen ganger	Ofte	Alltid
Er det en kamp å få barnet ditt til å spise?	(1) ❑	(2) ❑	(3) ❑	(4) ❑	(5) ❑
Blir du urolig hvis barnet ditt spiser for mye?	(1) ❑	(2) ❑	(3) ❑	(4) ❑	(5) ❑
Gir du barnet ditt mat for å unngå at det skal bli urolig, selv om du ikke tror det er sultent?	(1) ❑	(2) ❑	(3) ❑	(4) ❑	(5) ❑

Hvor enig er du i påstandene under?

	Svært uenig	Litt uenig	Hverken-eller	Litt enig	Svært enig
Hvis jeg ikke oppmuntrer barnet mitt til å spise, ville hun/han ikke spist nok	(1) ❑	(2) ❑	(3) ❑	(4) ❑	(5) ❑
Å gi barnet mitt mat er den beste måten å roe det på	(1) ❑	(2) ❑	(3) ❑	(4) ❑	(5) ❑
Jeg vet når barnet mitt er sultent	(1) ❑	(2) ❑	(3) ❑	(4) ❑	(5) ❑
Jeg er bekymret for at barnet mitt skal bli undervektig	(1) ❑	(2) ❑	(3) ❑	(4) ❑	(5) ❑
Jeg vet når barnet mitt er mett	(1) ❑	(2) ❑	(3) ❑	(4) ❑	(5) ❑
Barnet mitt vet når hun/han er sulten	(1) ❑	(2) ❑	(3) ❑	(4) ❑	(5) ❑
Jeg er bekymret for at barnet mitt skal bli overvektig	(1) ❑	(2) ❑	(3) ❑	(4) ❑	(5) ❑
Barnet mitt vet når hun/han er mett	(1) ❑	(2) ❑	(3) ❑	(4) ❑	(5) ❑

Hvor trygg føler du deg på følgende:

	Svært utrygg	Noe utrygg	Hverken-eller	Noe trygg	Svært trygg
At den maten du gir barnet ditt er sunn	(1) ❑	(2) ❑	(3) ❑	(4) ❑	(5) ❑

	Svært utrygg	Noe utrygg	Hverken-eller	Noe trygg	Svært trygg
At du kan få barnet ditt til å spise nok	(1) ❑	(2) ❑	(3) ❑	(4) ❑	(5) ❑
At du kan få barnet ditt til å prøve grønnsaker	(1) ❑	(2) ❑	(3) ❑	(4) ❑	(5) ❑
At du gir barnet ditt riktig mengde mat	(1) ❑	(2) ❑	(3) ❑	(4) ❑	(5) ❑
At du kan få barnet ditt til å smake på ny mat	(1) ❑	(2) ❑	(3) ❑	(4) ❑	(5) ❑

Her følger noen spørsmål om barnets generelle væremåte.
Tenk på hvordan dere vanligvis pleier å ha det når du svarer på spørsmålene:

Kryss av i hvilken grad du er enig eller uenig i følgende påstander om barnets humør og temperament.

	Svært uenig	Uenig	Noe uenig	Hverken enig eller uenig	Noe enig	Enig	Svært enig
Barnet sutrer og gråter mye	(1) ❑	(2) ❑	(3) ❑	(4) ❑	(5) ❑	(6) ❑	(7) ❑
Barnet er vanligvis lett å roe når hun/han gråter	(1) ❑	(2) ❑	(3) ❑	(4) ❑	(5) ❑	(6) ❑	(7) ❑
Det skal lite til før barnet er oppskaket og begynner å gråte	(1) ❑	(2) ❑	(3) ❑	(4) ❑	(5) ❑	(6) ❑	(7) ❑
Når barnet gråter, skriker hun/han vanligvis hissig og kraftig	(1) ❑	(2) ❑	(3) ❑	(4) ❑	(5) ❑	(6) ❑	(7) ❑
Hun/han er grei og lett å ha med å gjøre	(1) ❑	(2) ❑	(3) ❑	(4) ❑	(5) ❑	(6) ❑	(7) ❑

Kryss av om du er enig eller uenig i følgende påstander om barnets humør og temperament.

	Svært uenig	Uenig	Noe uenig	Hverken enig eller uenig	Litt enig	Enig	Svært enig
Barnet krever svært mye oppmerksomhet	(1) ❑	(2) ❑	(3) ❑	(4) ❑	(5) ❑	(6) ❑	(7) ❑
Når barnet overlates til seg selv, leker hun/han vanligvis fint med seg selv	(1) ❑	(2) ❑	(3) ❑	(4) ❑	(5) ❑	(6) ❑	(7) ❑
Barnet er såpass krevende at hun/han ville representere et betydelig problem for de fleste foreldre	(1) ❑	(2) ❑	(3) ❑	(4) ❑	(5) ❑	(6) ❑	(7) ❑
Barnet smiler og ler ofte	(1) ❑	(2) ❑	(3) ❑	(4) ❑	(5) ❑	(6) ❑	(7) ❑
Barnet er lett å legge og sovner fort	(1) ❑	(2) ❑	(3) ❑	(4) ❑	(5) ❑	(6) ❑	(7) ❑

Til slutt i denne delen kommer noen spørsmål om barneoppdragelse:

Hvor enig er du i påstandene under?

	Svært enig	Litt enig	Hverken-eller	Litt uenig	Svært uenig
Du kan skjemme bort en baby	(1) ❑	(2) ❑	(3) ❑	(4) ❑	(5) ❑
Barnet mitt trenger å lære forskjell på riktig og galt	(1) ❑	(2) ❑	(3) ❑	(4) ❑	(5) ❑
Små barn bør oppmuntres til å underholde seg selv	(1) ❑	(2) ❑	(3) ❑	(4) ❑	(5) ❑
Det er svært viktig at barnet mitt når utviklingsmessige milepæler til riktig tid	(1) ❑	(2) ❑	(3) ❑	(4) ❑	(5) ❑
Jeg har en fast dagsrytme for barnet mitt	(1) ❑	(2) ❑	(3) ❑	(4) ❑	(5) ❑
Jeg deltar på mange organiserte aktiviteter sammen med barnet mitt	(1) ❑	(2) ❑	(3) ❑	(4) ❑	(5) ❑

	Svært enig	Litt enig	Hverken-eller	Litt uenig	Svært uenig
Det er aldri for tidlig å begynne oppdragelsen av et barn	(1) ❑	(2) ❑	(3) ❑	(4) ❑	(5) ❑
Noen ganger gråter barnet mitt for å prøve og manipulere meg	(1) ❑	(2) ❑	(3) ❑	(4) ❑	(5) ❑

Hvor enig er du i påstandene under?

	Svært enig	Litt enig	Hverken-eller	Litt uenig	Svært uenig
Små barn trenger rutiner	(1) ❑	(2) ❑	(3) ❑	(4) ❑	(5) ❑
Jeg spør regelmessig andre mennesker om råd vedrørende atferden til barnet mitt	(1) ❑	(2) ❑	(3) ❑	(4) ❑	(5) ❑
Jeg passer på å leke, lese eller synge for barnet mitt jevnlig	(1) ❑	(2) ❑	(3) ❑	(4) ❑	(5) ❑
Jeg passer på å legge barnet mitt ned regelmessig	(1) ❑	(2) ❑	(3) ❑	(4) ❑	(5) ❑
Barnet mitt gjør noen ganger slemme ting	(1) ❑	(2) ❑	(3) ❑	(4) ❑	(5) ❑
Jeg synes mennesker som ikke lager seg rutiner gjør det vanskelig for seg selv.	(1) ❑	(2) ❑	(3) ❑	(4) ❑	(5) ❑
Jeg engster meg mye for barnet mitt	(1) ❑	(2) ❑	(3) ❑	(4) ❑	(5) ❑
Å stadig kose med barna gjør dem for avhengige	(1) ❑	(2) ❑	(3) ❑	(4) ❑	(5) ❑

Hvor enig er du i påstandene under?

	Svært enig	Litt enig	Hverken-eller	Litt uenig	Svært uenig
Barnet mitt lager seg egne rutiner	(1) ❑	(2) ❑	(3) ❑	(4) ❑	(5) ❑

	Svært enig	Litt enig	Hverken-eller	Litt uenig	Svært uenig
Jeg oppmuntrer barnet mitt til å utvikle nye ferdigheter som å krype eller lage lyder	(1) ❑	(2) ❑	(3) ❑	(4) ❑	(5) ❑
Jeg slår ofte opp i bøker eller liknende for å sjekke at barnet mitt er der det skal være	(1) ❑	(2) ❑	(3) ❑	(4) ❑	(5) ❑
Generelt så liker jeg å ha barnet mitt så nær meg som mulig	(1) ❑	(2) ❑	(3) ❑	(4) ❑	(5) ❑
Alle har det best når barnet følger en fast rytme	(1) ❑	(2) ❑	(3) ❑	(4) ❑	(5) ❑
Jeg oppsøker jevnlig helsesøster eller fastlege for råd vedrørende barnet mitt	(1) ❑	(2) ❑	(3) ❑	(4) ❑	(5) ❑
Barn under ett år trenger ikke disiplin	(1) ❑	(2) ❑	(3) ❑	(4) ❑	(5) ❑
Rutiner gjør barnet rolig og trygt	(1) ❑	(2) ❑	(3) ❑	(4) ❑	(5) ❑
Barn trenger mye stimuli fra sine foreldre som lek, lesing eller andre aktiviteter.	(1) ❑	(2) ❑	(3) ❑	(4) ❑	(5) ❑

Nå kommer andre del av spørreskjemaet med spørsmål om deg som er mor eller far. Spørsmålene omhandler bakgrunnsopplysninger, matvaner og vurdering av egen fysisk og psykisk helse.

Hva er din fødselsdato?
Må skrives dag.måned.år. Feks 22.12.2015

____

Hvilken sivilstand har du nå?

(1) ❑ Gift

(2) ❑ Samboer
(3) ❑ Enslig
(4) ❑ Skilt/separert
(5) ❑ Enke/enkemann
(6) ❑ Annet, beskriv _____

Hvor mange personer er det totalt i din husholdning?

Antall voksne _____

Antall barn _____

Alder på barn som ikke deltar i undersøkelsen
svar i hele år og komma mellom hvert barn; f.eks. 3, 5

_____

Er du, ev. mor til barnet som deltar i undersøkelsen, gravid på ny?

(1) ❑ Ja
(2) ❑ Nei

Har du eller barnets andre forelder et annet morsmål enn norsk?

(1) ❑ Ja
(2) ❑ Nei

Hvilket morsmål, beskriv

(1) ❑ Mor _____
(2) ❑ Far _____

Har en av barnets besteforeldre et annet morsmål enn norsk?

(1) ❑ Ja
(2) ❑ Nei

Hvilket morsmål, beskriv

(1) ❑ Mormor ______

(2) ❑ Morfar ______

(3) ❑ Farmor ______

(4) ❑ Farfar ______

Hvilken utdannelse har du?

Velg høyeste fullførte utdanning

(1) ❑ Mindre enn 9/10 års grunnskole

(2) ❑ Grunnskole

(3) ❑ Videregående skole

(4) ❑ Videregående yrkesfag

(5) ❑ Universitet/høyskole inntil 4 år

(6) ❑ Universitet/høyskole mer enn 4 år

(8) ❑ Annen utdanning

Hva er din hovedaktivitet?

Ev. hva var din hovedaktivitet før du ble gravid?

(1) ❑ Arbeid heltid

(2) ❑ Arbeid deltid

(3) ❑ Hjemmeværende

(4) ❑ Sykemeldt

(5) ❑ Permisjon

(6) ❑ Uføretrygdet

(7) ❑ Under attføring/rehabilitering

(8) ❑ Student/skoleelev

(9) ❑ Arbeidsledig

(10) ❑ Annet

Hvilket fylke bor du i?

(1) ❑ Akershus

(2) ❑ Aust-Agder

(3) ❑ Buskerud

(4) ❑ Finnmark

(5) ❑ Hedmark
(6) ❑ Hordaland
(7) ❑ Møre og Romsdal
(8) ❑ Nord-Trøndelag
(9) ❑ Nordland
(10) ❑ Oppland
(11) ❑ Oslo
(12) ❑ Rogaland
(13) ❑ Sogn og Fjordane
(14) ❑ Sør-Trøndelag
(15) ❑ Telemark
(16) ❑ Troms
(18) ❑ Vest-Agder
(17) ❑ Vestfold
(19) ❑ Østfold

Cirka hvor mange innbyggere bor på ditt nærmeste tettsted?

(1) ❑ 0-4999
(2) ❑ 5000-14999
(3) ❑ 15000-49999
(4) ❑ Over 50000

Eier du/dere egen bolig?

(1) ❑ Ja
(2) ❑ Nei

Er økonomien slik at du/dere vil klare en uforutsett utgift på ca 3000 kr, f.eks. til tannlege eller reperasjon?

(1) ❑ Ja
(2) ❑ Nei
(3) ❑ Vet ikke

Har det i løpet av det siste halve året hendt at du/dere har hatt vansker med å klare løpende utgifter til mat, transport, husleie og liknende?

(1) ❑ Nei, aldri

(2) ❑ Ja, en sjelden gang
(3) ❑ Ja, av og til
(4) ❑ Ja, ofte

Nå følger noen spørsmål om levevaner og livsstil:

Hvor høy er du?
Svar i antall centimeter

______

Hvor mye veier du nå?
Svar i antall kg

______

Prøver du å slanke deg?

(1) ❑ Nei, vekten min er passe
(2) ❑ Nei, jeg trenger å gå opp i vekt
(3) ❑ Nei, men jeg trenger å gå ned i vekt
(4) ❑ Ja

Røyker du?
(1) ❑ Nei, har aldri røykt regelmessig
(2) ❑ Nei, har sluttet
(3) ❑ Ja, men ikke daglig
(4) ❑ Ja, daglig

Snuser du?
(1) ❑ Nei, har aldri snust regelmessig
(2) ❑ Nei, har sluttet
(3) ❑ Ja, men ikke daglig

(4) ❑ Ja, daglig

Hvor mange ganger i uken er du så fysisk aktiv at du blir andpusten eller svett nå for tiden?
Tilsammen minst 30 min. per gang

(1) ❑ Aldri
(2) ❑ Mindre enn 1 gang per uke
(3) ❑ 1 gang per uke
(4) ❑ 2 ganger per uke
(5) ❑ 3-4 ganger per uke
(6) ❑ 5 ganger eller mer per uke

På fritiden; omtrent hvor mye tid bruker du tilsammen ved en TV, PC/nettbrett eller smarttelefon?

(1) ❑ Mindre enn en time daglig
(2) ❑ Mellom 1 og 2 timer daglig
(3) ❑ Mellom 2 og 4 timer daglig
(4) ❑ Mellom 4 og 6 timer daglig
(5) ❑ Mer enn 6 timer daglig

Nå følger noen spørsmål om kost og matvaner.
Vi spør om dine spisevaner slik de vanligvis er. Vi er klar over at kostholdet varierer fra dag til dag, prøv derfor så godt du kan å gi et "gjennomsnitt" av dine spisevaner slik de har vært det siste året.

Hvordan vil du beskrive ditt eget kosthold?

(1) ❑ Jeg har et vanlig variert kosthold
(2) ❑ Jeg spiser ikke fisk
(3) ❑ Jeg spiser ikke kjøtt
(4) ❑ Jeg er vegetarianer
(5) ❑ Jeg følger en spesiell diett, beskriv: ______
(6) ❑ Ingen av beskrivelsene passer

Hvor mange ganger pleier du å spise følgende måltider i løpet av en uke?

	Aldri/sjelden	1 g/u	2 g/u	3 g/u	4 g/u	5 g/u	6 g/u	Hver dag
Frokost	(1) ❑	(2) ❑	(3) ❑	(4) ❑	(5) ❑	(6) ❑	(7) ❑	(8) ❑
Formiddagsmat/lunsj	(1) ❑	(2) ❑	(3) ❑	(4) ❑	(5) ❑	(6) ❑	(7) ❑	(8) ❑
Mellommåltid før middag	(1) ❑	(2) ❑	(3) ❑	(4) ❑	(5) ❑	(6) ❑	(7) ❑	(8) ❑
Middag	(1) ❑	(2) ❑	(3) ❑	(4) ❑	(5) ❑	(6) ❑	(7) ❑	(8) ❑
Mellommåltid etter middag	(1) ❑	(2) ❑	(3) ❑	(4) ❑	(5) ❑	(6) ❑	(7) ❑	(8) ❑
Kveldsmat	(1) ❑	(2) ❑	(3) ❑	(4) ❑	(5) ❑	(6) ❑	(7) ❑	(8) ❑
Andre mellommåltider, snack el.l.	(1) ❑	(2) ❑	(3) ❑	(4) ❑	(5) ❑	(6) ❑	(7) ❑	(8) ❑

Har du hovedansvaret for matlagingen hjemme?

(1) ❑ Ja

(2) ❑ Nei

(3) ❑ Ansvaret er delt

Hvor ofte gjør du følgende?

	Aldri	Mindre enn 1 g/u	1 g/u	2 g/u	3 g/u	4 g/u	5 g/u	6 g/u	Hver dag
Kutter opp grønnsaker	(1) ❑	(2) ❑	(3) ❑	(4) ❑	(5) ❑	(6) ❑	(7) ❑	(8) ❑	(9) ❑
Kutter opp frukt	(1) ❑	(2) ❑	(3) ❑	(4) ❑	(5) ❑	(6) ❑	(7) ❑	(8) ❑	(9) ❑
Lager middag fra bunnen	(1) ❑	(2) ❑	(3) ❑	(4) ❑	(5) ❑	(6) ❑	(7) ❑	(8) ❑	(9) ❑

Spiser du hurtigmat (fra gatekjøkken, bensinstasjon o.l.) mer enn 1 gang per uke?

(1) ❑ ja

(2) ❑ nei

Hvor mye drikker du vanligvis av følgende drikker?
En enhet er det samme som et glass eller en kopp

	Drikker aldri/sjelden	1-3 enhet/mnd.	1-3 enhet/uke	4-6 enheter/uke	1-3 enhet/dag	4-6 enheter/dag	7 enheter eller fler daglig
Helmelk	(1) ❑	(2) ❑	(3) ❑	(4) ❑	(5) ❑	(6) ❑	(7) ❑
Skummet-, lett- eller ekstralett melk	(1) ❑	(2) ❑	(3) ❑	(4) ❑	(5) ❑	(6) ❑	(7) ❑
Appelsinjuice/fruktjuice	(1) ❑	(2) ❑	(3) ❑	(4) ❑	(5) ❑	(6) ❑	(7) ❑
Saft med sukker	(1) ❑	(2) ❑	(3) ❑	(4) ❑	(5) ❑	(6) ❑	(7) ❑
Saft, kunstig søtet	(1) ❑	(2) ❑	(3) ❑	(4) ❑	(5) ❑	(6) ❑	(7) ❑

Hvor mye drikker du vanligvis av følgende drikker?
En enhet er det samme som et glass eller en kopp

	Drikker aldri/sjelden	1-3 enhet/mnd.	1-3 enhet/uke	4-6 enheter/uke	1-3 enhet/dag	4-6 enheter/dag	7 eller fler enheter daglig
Brus med sukker	(1) ❑	(2) ❑	(3) ❑	(4) ❑	(5) ❑	(6) ❑	(7) ❑
Lettbrus, kunstig søtet	(1) ❑	(2) ❑	(3) ❑	(4) ❑	(5) ❑	(6) ❑	(7) ❑
Kaffe	(1) ❑	(2) ❑	(3) ❑	(4) ❑	(5) ❑	(6) ❑	(7) ❑
Te	(1) ❑	(2) ❑	(3) ❑	(4) ❑	(5) ❑	(6) ❑	(7) ❑
Øl	(1) ❑	(2) ❑	(3) ❑	(4) ❑	(5) ❑	(6) ❑	(7) ❑
Vin	(1) ❑	(2) ❑	(3) ❑	(4) ❑	(5) ❑	(6) ❑	(7) ❑

Hvor ofte spiser du følgende matvarer?

	Aldri/sjelden	1-3 ganger/måned	1-3 ganger/uke	4-6 ganger/uke	1 gang/dag	2 ganger/dag	3 ganger/dag	4 ganger eller fler/dag
Kokte poteter	(1) ❑	(2) ❑	(4) ❑	(5) ❑	(6) ❑	(7) ❑	(8) ❑	(9) ❑
Pommes frites	(1) ❑	(2) ❑	(4) ❑	(5) ❑	(6) ❑	(7) ❑	(8) ❑	(9) ❑
Ris	(1) ❑	(2) ❑	(4) ❑	(5) ❑	(6) ❑	(7) ❑	(8) ❑	(9) ❑
Pasta	(1) ❑	(2) ❑	(4) ❑	(5) ❑	(6) ❑	(7) ❑	(8) ❑	(9) ❑
Fullkornspasta	(1) ❑	(2) ❑	(4) ❑	(5) ❑	(6) ❑	(7) ❑	(8) ❑	(9) ❑
Rå grønnsaker/salater	(1) ❑	(2) ❑	(4) ❑	(5) ❑	(6) ❑	(7) ❑	(8) ❑	(9) ❑

	Aldri/sjelden	1-3 ganger/måned	1-3 ganger/uke	4-6 ganger/uke	1 gang/dag	2 ganger/dag	3 ganger/dag	4 ganger eller fler/dag
Kokte grønnsaker	(1) ❑	(2) ❑	(4) ❑	(5) ❑	(6) ❑	(7) ❑	(8) ❑	(9) ❑

Hvor ofte spiser du følgende matvarer?

	Aldri/sjelden	1-3 ganger/måned	1-3 ganger/uke	4-6 ganger/uke	1 gang/dag	2 ganger/dag	3 ganger/dag	4 ganger eller fler/dag
Fisk til middag (kokt eller stekt)	(1) ❑	(2) ❑	(4) ❑	(5) ❑	(6) ❑	(7) ❑	(8) ❑	(9) ❑
Fiskekaker/fiskeboller	(1) ❑	(2) ❑	(4) ❑	(5) ❑	(6) ❑	(7) ❑	(8) ❑	(9) ❑
Retter med kjøttdeig	(1) ❑	(2) ❑	(4) ❑	(5) ❑	(6) ❑	(7) ❑	(8) ❑	(9) ❑
Rent kjøtt	(1) ❑	(2) ❑	(4) ❑	(5) ❑	(6) ❑	(7) ❑	(8) ❑	(9) ❑
Kylling/kalkun	(1) ❑	(2) ❑	(4) ❑	(5) ❑	(6) ❑	(7) ❑	(8) ❑	(9) ❑
Pizza	(1) ❑	(2) ❑	(4) ❑	(5) ❑	(6) ❑	(7) ❑	(8) ❑	(9) ❑
Pølser/hamburger	(1) ❑	(2) ❑	(4) ❑	(5) ❑	(6) ❑	(7) ❑	(8) ❑	(9) ❑

Hvor ofte spiser du følgende matvarer?

	Aldri/sjelden	1-3 ganger/måned	1-3 ganger/uke	4-6 ganger/uke	1 gang/dag	2 ganger/dag	3 ganger/dag	4 ganger eller fler/dag
Frukt	(1) ❑	(2) ❑	(4) ❑	(5) ❑	(6) ❑	(7) ❑	(8) ❑	(9) ❑
Bær	(1) ❑	(2) ❑	(4) ❑	(5) ❑	(6) ❑	(7) ❑	(8) ❑	(9) ❑
Grovbrød (over 50% sammalt mel)	(1) ❑	(2) ❑	(4) ❑	(5) ❑	(6) ❑	(7) ❑	(8) ❑	(9) ❑
Fint brød/loff	(1) ❑	(2) ❑	(4) ❑	(5) ❑	(6) ❑	(7) ❑	(8) ❑	(9) ❑
Kaker, kjeks o.l.	(1) ❑	(2) ❑	(4) ❑	(5) ❑	(6) ❑	(7) ❑	(8) ❑	(9) ❑
Desserter, iskrem o.l.	(1) ❑	(2) ❑	(4) ❑	(5) ❑	(6) ❑	(7) ❑	(8) ❑	(9) ❑
Godterier	(1) ❑	(2) ❑	(4) ❑	(5) ❑	(6) ❑	(7) ❑	(8) ❑	(9) ❑
Sjokolade	(1) ❑	(2) ❑	(4) ❑	(5) ❑	(6) ❑	(7) ❑	(8) ❑	(9) ❑
Potetgull o.l.	(1) ❑	(2) ❑	(4) ❑	(5) ❑	(6) ❑	(7) ❑	(8) ❑	(9) ❑
Peanøtter	(1) ❑	(2) ❑	(4) ❑	(5) ❑	(6) ❑	(7) ❑	(8) ❑	(9) ❑

Bruker du noen form for kost-tilskudd?

	Ja	Nei
Multivitamin-/vitamintilskudd	(1) ❑	(2) ❑
Tran/fiskeolje	(1) ❑	(2) ❑

Hvor enig er du i følgende?

	Svært uenig	Moderat uenig	Litt uenig	Verken enig eller uenig	Litt enig	Moderat enig	Svært enig
Jeg prøver stadig ny og ulik type mat	(1) ❑	(5) ❑	(7) ❑	(6) ❑	(8) ❑	(9) ❑	(4) ❑
Jeg stoler ikke på ukjent mat	(1) ❑	(5) ❑	(7) ❑	(6) ❑	(8) ❑	(9) ❑	(4) ❑
Hvis jeg ikke vet hva som er i maten, vil jeg ikke smake	(1) ❑	(5) ❑	(7) ❑	(6) ❑	(8) ❑	(9) ❑	(4) ❑
Jeg liker mat fra forskjellige land	(1) ❑	(5) ❑	(7) ❑	(6) ❑	(8) ❑	(9) ❑	(4) ❑
Etnisk mat ser for merkelig ut til å spises	(1) ❑	(5) ❑	(7) ❑	(6) ❑	(8) ❑	(9) ❑	(4) ❑
I middagsselskaper prøver jeg nye retter	(1) ❑	(5) ❑	(7) ❑	(6) ❑	(8) ❑	(9) ❑	(4) ❑
Jeg er redd for å spise noe jeg ikke har spist før	(1) ❑	(5) ❑	(7) ❑	(6) ❑	(8) ❑	(9) ❑	(4) ❑
Jeg er veldig kresen på hva slags mat jeg vil spise	(1) ❑	(5) ❑	(7) ❑	(6) ❑	(8) ❑	(9) ❑	(4) ❑
Jeg spiser nesten all slags mat	(1) ❑	(5) ❑	(7) ❑	(6) ❑	(8) ❑	(9) ❑	(4) ❑
Jeg liker å prøve etniske restauranter	(1) ❑	(5) ❑	(7) ❑	(6) ❑	(8) ❑	(9) ❑	(4) ❑

Til slutt kommer noen spørsmål om opplevelsen av egen fysisk og psykisk helse:

Alt i alt; hvordan vil du karakterisere din fysiske helse?

(1) ❑ Meget god
(5) ❑ God
(6) ❑ Dårlig
(7) ❑ Svært dårlig

I hvilken grad begrenser din helse dine hverdagslige gjøremål?

(1) ❑ I stor grad
(2) ❑ I noen grad
(3) ❑ I liten grad
(4) ❑ Ikke i det hele tatt

Har du i løpet av de siste to ukene vært plaget med noe av det følgende?

	Ikke plaget	Litt plaget	Ganske mye plaget	Veldig mye plaget
Stadig redd eller engstelig	(1) ❑	(2) ❑	(3) ❑	(4) ❑
Nervøsitet og indre uro	(1) ❑	(2) ❑	(3) ❑	(4) ❑
Følelse av håpløshet med hensyn til fremtiden	(1) ❑	(2) ❑	(3) ❑	(4) ❑
Nedtrykt, tungsindig	(1) ❑	(2) ❑	(3) ❑	(4) ❑
Mye bekymret eller urolig	(1) ❑	(2) ❑	(3) ❑	(4) ❑
Følelse av at alt er et slit	(1) ❑	(2) ❑	(3) ❑	(4) ❑
Føler deg anspent eller oppjaget	(1) ❑	(2) ❑	(3) ❑	(4) ❑
Plutselig frykt uten grunn	(1) ❑	(2) ❑	(3) ❑	(4) ❑

Hvor ofte opplever du følgende i ditt daglige liv?

	Sjelden/aldri	Nokså sjelden	Noen ganger	Ofte	Veldig ofte
Føler deg glad for noe	(1) ❑	(2) ❑	(3) ❑	(4) ❑	(5) ❑
Føler deg lykkelig	(1) ❑	(2) ❑	(3) ❑	(4) ❑	(5) ❑
Føler deg oppstemt, som om alt legger seg tilrette for deg	(1) ❑	(2) ❑	(3) ❑	(4) ❑	(5) ❑
Føler at du vil skrike til noen	(1) ❑	(2) ❑	(3) ❑	(4) ❑	(5) ❑

	Sjelden/aldri	Nokså sjelden	Noen ganger	Ofte	Veldig ofte
eller slå løs på ting					
Føler deg sint, irritert eller ergelig	(1) ❑	(2) ❑	(3) ❑	(4) ❑	(5) ❑
Føler deg rasende på noen	(1) ❑	(2) ❑	(3) ❑	(4) ❑	(5) ❑

Hvor riktige er disse utsagnene for deg?

	Ikke riktig	Litt riktig	Nesten riktig	Helt riktig
Jeg klarer alltid å løse vanskelige problemer hvis jeg prøver hardt nok	(1) ❑	(2) ❑	(3) ❑	(4) ❑
Hvis noen motarbeider meg, finner jeg en måte å oppnå det jeg vil på	(1) ❑	(2) ❑	(3) ❑	(4) ❑
Jeg er sikker på at jeg kan mestre uventede hendelser	(1) ❑	(2) ❑	(3) ❑	(4) ❑
Jeg er rolig når jeg møter vanskeligheter, fordi jeg stoler på min evne til å klare meg	(1) ❑	(2) ❑	(3) ❑	(4) ❑
Dersom jeg er i en knipe, finner jeg vanligvis en løsning	(1) ❑	(2) ❑	(3) ❑	(4) ❑

Da er du ferdig med å fylle ut skjemaet.
Du sender det inn ved å trykke på knappen under.

Tusen takk!
